# A dynamical systems treatment of transcriptomic trajectories in hematopoiesis

**DOI:** 10.1242/dev.201280

**Published:** 2023-06-01

**Authors:** Simon L. Freedman, Bingxian Xu, Sidhartha Goyal, Madhav Mani

**Affiliations:** ^1^ Illumina, San Diego, CA 92122, USA; ^2^ NSF-Simons Center for Quantitative Biology, Northwestern University, Evanston, IL 60208, USA; ^3^ Department of Molecular Biosciences, Northwestern University, Evanston, IL 60208, USA; ^4^ Department of Physics, University of Toronto, Toronto, ON M5R 2M8, Canada; ^5^ Institute of Biomedical Engineering, University of Toronto, Toronto, ON M5R 2M8, Canada; ^6^ Department of Engineering Sciences and Applied Mathematics, Northwestern University, Evanston, IL 60208, USA

**Keywords:** Differentiation, Bifurcation, Single-cell RNA-seq, Pseudotime, Waddington

## Abstract

Inspired by Waddington's illustration of an epigenetic landscape, cell-fate transitions have been envisioned as bifurcating dynamical systems, wherein exogenous signaling dynamics couple to the enormously complex signaling and transcriptional machinery of a cell to elicit qualitative transitions in its collective state. Single-cell RNA sequencing (scRNA-seq), which measures the distributions of possible transcriptional states in large populations of differentiating cells, provides an alternate view, in which development is marked by the variations of a myriad of genes. Here, we present a mathematical formalism for rigorously evaluating, from a dynamical systems perspective, whether scRNA-seq trajectories display statistical signatures consistent with bifurcations and, as a case study, pinpoint regions of multistability along the neutrophil branch of hematopoeitic differentiation. Additionally, we leverage the geometric features of linear instability to identify the low-dimensional phase plane in gene expression space within which the multistability unfolds, highlighting novel genetic players that are crucial for neutrophil differentiation. Broadly, we show that a dynamical systems treatment of scRNA-seq data provides mechanistic insights into the high-dimensional processes of cellular differentiation, taking a step toward systematic construction of mathematical models for transcriptomic dynamics.

## Teaser

**Highlighted Article:** Bridging the mathematical theory of dynamical systems and high-dimensional transcriptomic data of differentiating cells by studying statistical signatures of linear instability, encoded in a time-varying covariance matrix.

## INTRODUCTION

**Fig. 1.**
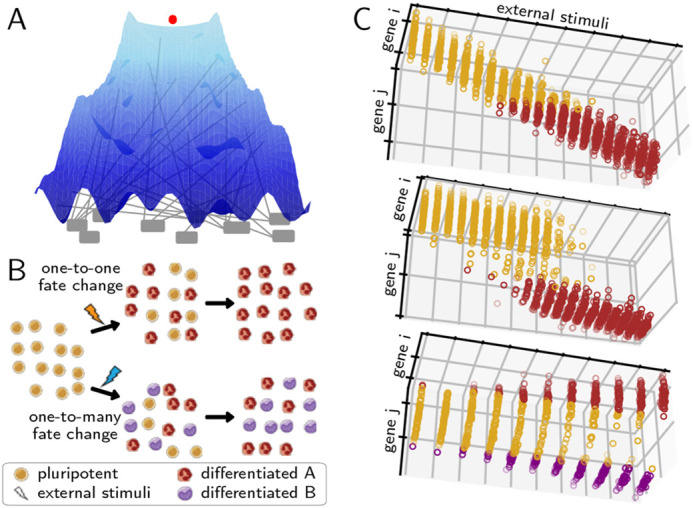
**Cell type differentiation as a dynamical process.** (A) Reimagination of Waddington's landscape of cell fate commitment in which cell fates are represented as valleys, commitment barriers as hills and gene activity as pegs underneath that control the heights of hills and valleys. (B) Schematic cell-population snapshots of maturation (top), in which one cell fate transitions to a different one, and a cell fate decision (bottom), in which a pluripotent cell differentiates to either of two lineages. Cell type images by A. Rad and M. Häggström. CC-BY-SA 3.0 license. (C) Gene expression trajectories for cells (dots) at varying levels of a differentiating stimuli for cases where the differentiation landscape does not bifurcate (top), undergoes a saddle-node bifurcation (middle) or undergoes a pitchfork bifurcation (bottom).

During development and tissue regeneration, it is envisioned that cells progress through multiple transitions to ultimately adopt a distinguishable function. Although each transition en route to a terminal fate involves the coordination of myriads of molecules and complex gene regulatory networks interacting with external factors, there is a common view that they depend on significantly fewer control parameters. This view was notably explicated by Conrad Waddington in an illustration of an epigenetic space as a tilted, bifurcating landscape, where a vast number of nodes (genes) provide the scaffold for the smooth hills and valleys (cell state) down which a pebble (cell) can reliably roll until it finds a resting position (terminal fate) (Fig. 1A) (Waddington, 1957).

Many of the characteristics of Waddington's landscape have been codified into the language of dynamical systems, including that cell fates resemble valleys (attractors) in gene expression or transciptomic space (Huang et al., 2005; Corson and Siggia, 2012; Slack et al., 1991; Camacho-Aguilar et al., 2021), that a small amount of stable states can emerge from large interconnected Boolean networks (Kauffman, 1969), and that known genetic interactions can yield multiple cell fates (bistability) (Huang et al., 2007; Weston et al., 2018). Waddington's illustration has also motivated analysis of the wealth of data captured in single-cell RNA-sequencing (scRNA-seq), in which the transcriptome of individual cells are measured, often at multiple time-points, as they differentiate. For example, fitting a mathematical model of a pitchfork bifurcation to scRNA-seq data yields predictions for developmental perturbations (Marco et al., 2014), reducing the dimensionality of large transcriptomic matrices can enhance the resolution of bifurcations to precisely determine the genes enabling a cell fate decision (Setty et al., 2016; Tusi et al., 2018), and well characterized cell-lineage relationships can be used to extract predictive models of gene regulation (Furchtgott et al., 2017; Qiu et al., 2020; Wang et al., 2022). While these studies generally characterize cell fate decisions as bifurcations of an underlying developmental landscape, other studies formulate cell fate transitions as stochastic jumps between co-existing states of a multimodal cell-fate landscape that can occur even in the absence of bifurcations, to infer lineage relationships and state transition probabilities (Weinreb et al., 2018a; Zhou et al., 2021; Lange et al., 2022).

In the face of these contrasting views, it remains unclear when, during development, transcriptomes undergo bifurcations and whether they can be identified purely from statistical analyses of single-cell expression data alone. To address these unknowns, we note that as a bifurcation is a qualitative augmentation of the steady state solutions, or branches, of a dynamical system that occurs as a control parameter varies, detecting bifurcations from transcriptomic data requires that steady states and control parameters exist, and that their dynamics can be identified from the data. We hypothesized an association between cell fates and transcriptomic steady states in scRNA-seq data, as the dynamic molecular processes that lead to transcriptomic changes, such as signal transduction and transcription, generally occur in the order of seconds and minutes (Shamir et al., 2016), whereas cell fates change over the course of hours or days (Slack et al., 1991), yielding a significant separation between the time scales of molecular mechanisms and data collection. We also hypothesized that inferred developmental time (pseudotime) could be used as a high resolution readout of a biological control parameter to pinpoint developmental bifurcations, as it coincides well with intrinsic cellular dynamics, and may therefore correlate with known biological control parameters, such as morphogen concentration (Trapnell et al., 2014; Setty et al., 2016; Street et al., 2018).

Here, we use these hypotheses to lay out and demonstrate a statistical formalism for detecting and interrogating bifurcations in developmental fate transitions directly from transcriptomic pseudotime trajectories. Contrasting previous studies (Marco et al., 2014; Setty et al., 2016; Huang et al., 2007; Weston et al., 2018), we do not assume any specific mathematical form for the underlying genetic interactions, nor do we assume the shape, or even existence, of an underlying cell-fate landscape (Fig. 1B) (Chen et al., 2012; Mojtahedi et al., 2016) as it is not our goal to discern a specific model. Instead we rigorously query whether the necessary statistical signatures of bifurcations are present in a developmental timecourse. We build on and compare with similar styles of approach, which use correlation structure to detect signatures and molecular mechanisms of disease (Chen et al., 2012; Liu et al., 2012), analyze differentiation processes in temporal and pseudotemporal gene expression trajectories (Mojtahedi et al., 2016; Chen et al., 2018), and characterize reversibility in saddle-node bifurcations (Li et al., 2019). We show that our dynamical systems-driven approach enables us to distinguish between three different types of transcriptomic variation directly from systems-level data: a non-bifurcative cell fate change that is due to continuous changes in gene expression (Fig. 1C, top); a cell fate change that is due to a one-to-one state transition (Fig. 1C, middle), such as those that may occur during terminal-fate maturation (Ferrell, 2012); and a cell fate change that is due to a one-to-many state transition, e.g. those that occur when pluripotent cells decide between multiple cell lineages (Fig. 1C, bottom). We apply our framework to a class of *in silico* high-dimensional genetic networks to demonstrate its ability to recover the salient features of a bifurcating dynamical system, and examine the effects of high dimensionality and noise. We demonstrate the utility of our framework in the context of a recently published scRNA-seq exploration of hematopoiesis (Weinreb et al., 2020), and show that cell-fate bifurcations can be pinpointed and analyzed in scRNA-seq data, even without detailed knowledge of the dynamics and controls of the underlying system. Finally, we demonstrate that our framework allows us to identify a low-dimensional phase plane in which the dynamics unfolds, and can be used to distinguish new cellular clusters and extract genetic relationships that are pivotal to the bifurcative cell-fate change.

## RESULTS

In this section, we show how the Continuous Time Lyapunov (CTL) equation (Appendix S1, section 1) can be used to investigate bifurcations in transcriptomic trajectories. An advantage of this framework is that we do not have to posit any specific functional form for the dynamical processes that yields a transcriptomic state or a shape, or even the existence of an underlying developmental landscape, only that the dynamical processes are (1) stochastic and Markovian (Rosenfeld et al., 2005; Raj and Van Oudenaarden, 2008; Gregor et al., 2007; Tkačik et al., 2008; Weinreb et al., 2018b; Zhou et al., 2021) ; and (2) occur at significantly faster timescales (seconds to minutes) than the timescales over which transitions in cellular fates are observed (hours to days) (Shamir et al., 2016; Slack et al., 1991). A consequence of these two assumptions (see details in the Materials and Methods section ‘Continuous time Lyapunov equation for transcriptomic matrices’) is that the local time evolution of the transcriptomic profile of a cell is controlled by a single matrix, the Jacobian (***J***), where 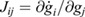 is the effect of the amount of gene *j* on the dynamics of gene *i*. Generically, the local geometry of a dynamical system can be obtained from its diagonalization, ***J***=***P***Λ***P***^−1^, where Λ is a diagonal matrix of eigenvalues 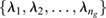 and ***P***^***T***^ is the square matrix of eigenvectors 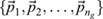. If the system has a single stable transcriptomic state, then 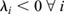, in the same way that highly convex curvature is associated with a single fixed point (see Appendix S1, section 1 and Fig. S1, for an example). Conversely, if the system is undergoing a bifurcation, than the largest Jacobian eigenvalue, which we refer to as *λ*_*d*_, and points in the 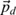 direction, must approach 0 from below, in the same way that flat curvature enables a fixed point exchange (Appendix S1, section 1). In the absence of a model, the eigenvalues and eigenvectors of the Jacobian are generally inaccessible from the CTL equation, even if the stochasticity is parameterized, as the covariance is a symmetric matrix and the Jacobian is asymmetric, yielding twice as many unknowns as there are equations. However, at a bifurcation, the CTL simplifies considerably, such that
(1)

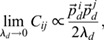
where *C*_*ij*_ is the covariance of gene *i* with gene *j* (Oku and Aihara, 2018). This simplification yields three key insights into the eigen-decomposition of the Jacobian directly from the eigen-decomposition of the covariance, 
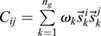
, where 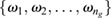 are its eigenvalues and 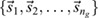 are its eigenvectors. First, as all 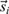, by definition, normalize to 1, for at least one covariance eigenvalue (*ω*_1_, without loss of generality):
(2)

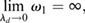
i.e. the covariance diverges along the principal direction 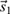. Second, it can be shown (see Materials and Methods section ‘Bifurcation eigenvector equivalence’) that
(3)

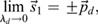
meaning the direction of maximal covariance is identical to the direction of the bifurcation! Third, a direct result of Eqn. 1 is that
(4)

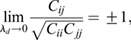
meaning the Pearson's correlation coefficient 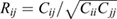 of the data along axes *i* and *j* becomes maximal, provided that their corresponding loadings on the eigen-vector (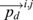) are non-zero.

The correlation structure and expansion of correlation coefficients at a bifurcation (Eqn. 4) has been used previously in the theory of Dynamical Network Biomarkers, or DNB (Chen et al., 2012), to explore bifurcations in cases where it can be determined which state variables are mechanistically involved in the bifurcation, including single-cell data (Mojtahedi et al., 2016). In our analysis, we analytically (Appendix S1, section 2) and empirically compare with this method, but note that unlike DNB analysis, it is not necessary to delineate which genes drive the bifurcation, as Eqn. 2 is a global feature of the covariance. Additionally, in contrast to previous studies that focus on correlation coefficients, we explore how covariance eigenvectors (Eqn. 3) provide direct insight into the underlying mechanisms driving developmental bifurcations.

Thus, three specific changes to the transcriptomic covariance data, Eqns. 2-4, that can be determined from observations of state variables, can inform us of the salient features of the system, its bifurcations, even when we have no direct access to the generative model for the dynamics or to its corresponding underlying geometry. Notably, these features rely only on the transcriptomic data being sampled from the vicinity of a steady state, and do not rely on special circumstances, such as the Jacobian being symmetric, or on the noise being of a particular nature. We first use theoretical models of noisy, high-dimensional genetic networks to demonstrate how this approach can be leveraged to detect and assess bifurcations of an underlying dynamical system from observations of state-variables alone (for a procedural outline, see Materials and Methods section ‘Analysis pipeline’). Following this, we emphasize the power of this approach by directly applying it to scRNA-seq data for the neutrophil lineage in the hematopoietic system.

### Covariance analysis recovers salient features of a high-dimensional *in silico* gene regulatory network

**Fig. 2.**
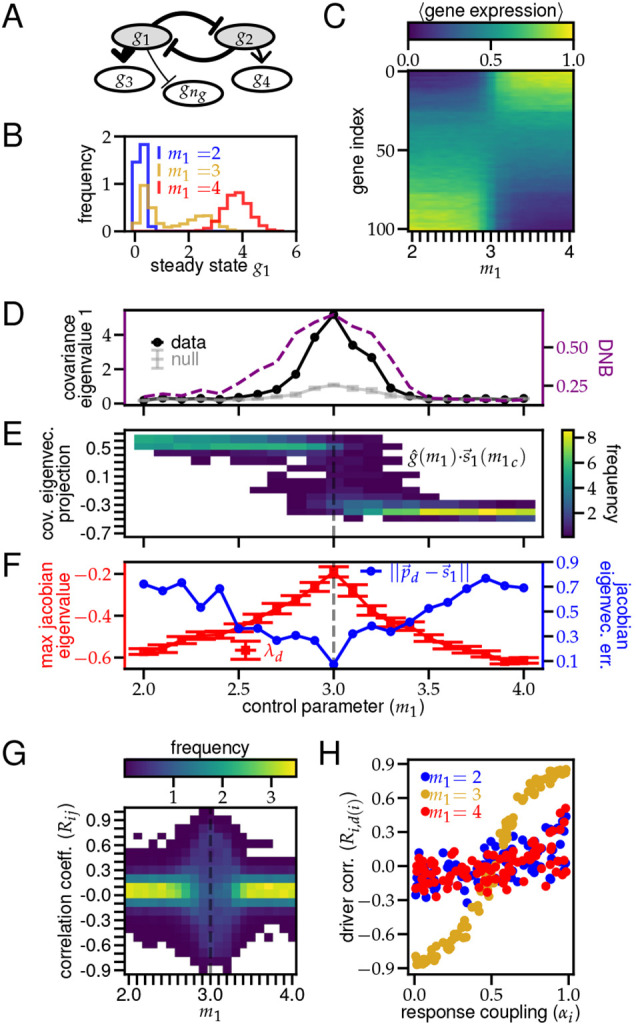
**Analysis of a gene regulatory network around a saddle-node bifurcation.** (A) Schematic of the GRN for 5 of the 102 genes. Undisplayed nodes have a unidirectional arrow stemming from either *g*_1_ or *g*_2_. (B) Distributions of *g*_1_ at steady state (see Materials and Methods section ‘Simulation methodology’) for three values of *m*_1_. (C-G) GRN observations as a function of the bifurcating variable *m*_1_ evaluated over a distribution of 100 cells (see Materials and Methods section ‘Simulation methodology’). (C) Average final expression for each gene. Expression of driver genes, *g*_1,2_ (bottom and top rows, respectively), are min-max normalized. Response genes are sorted by their corresponding driver [***d***(***i***)] and activation level (*α*_***i***_). (D) Black and gray indicate largest eigenvalue of covariance matrix shifted to have 0 min. Purple dashed line indicates DNB order parameter for gene expression matrix, where driver genes and tightly coupled responders |*α*_***i***_−0.5|<0.25 are in the DNB (Chen et al., 2012). (E) Distribution of normalized gene expression for each cell projected onto the bifurcating axis. (F) Red squares indicate the largest eigenvalue of the Jacobian matrix. Error bars are s.e.m. Blue circles indicate Euclidean distance from the corresponding Jacobian eigenvector (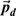) to the principle covariance eigenvector 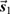. (G) Distribution of Pearson's correlation coefficients for all gene pairs. (H) Correlation coefficients between responding genes and their driver as a function of the coupling coefficient ***α*** (Eqn. 6) at three values of *m*_1_. Each column in E and G integrates to 1.

To better understand our mathematical framework in the context of scRNA-seq data, where the large number of discordant genes and biological noise may obfuscate the predicted covariance signal that is indicative of a bifurcation, and cell fate changes may take different geometric forms, we tested the framework on a noisy, high-dimensional, gene-regulatory network (GRN), illustrated in Fig. 2A. The deterministic aspects of this GRN are governed by a set of explicit ordinary differential equations, 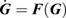, and stochasticity is incorporated by simulating the GRN with Poissonian noise (see Materials and Methods section ‘Simulation methodology’). In the GRN, cell fate transitions result from two mutually inhibiting ‘driver’ genes, *g*_1_ and *g*_2_, via their dynamics:
(5)

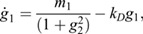
where *k*_*D*_ are their degradation rate, and *m*_1,2_ determine the scales of their synthesis (Gardner et al., 2000). Varying the control parameter *m*_1_ yields a saddle-node bifurcation in gene-expression while varying *k*_*D*_ yields a pitchfork bifurcation (see Appendix S1, section 3 and Fig. S2). Similar networks have been analyzed to provide insight into gene inhibition and activation (Gallivan et al., 2020), and into a diversity of biological systems, such as the lac-operon (Ozbudak et al., 2004) and cell-cycle control (Novak and Tyson, 1993).

As GRNs typically involve hundreds of genes, we include an additional *n*_*g*_−2 genes in the network that respond variably to one of the two driver genes, according to
(6)

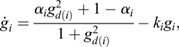
where *i*∈[3, *n*_*g*_], *k*_*i*_ is the degradation rate of *i*^th^ gene, *g*_*i*_ is its expression, *d*(*i*) indicates the driver (*d*(*i*)=1 if *g*_*i*_ responds to *g*_1_ and *d*(*i*)=2 if it responds to *g*_2_) and *α*_*i*_∈[0, 1] is the strength of the connection with its driver (*α*_*i*_=0 yields full inhibition and *α*_*i*_=1 yields full activation). Although this GRN can be made more complex, by including feedback from the responding genes to the two driver genes or by increasing the number of driver genes themselves, this simple model provides an interpretable demonstration of our proposed scheme.

We simulated this model for a fixed number of genes (*n*_*g*_), statistical replicates or cells (*n*_*c*_), noise scale (*s*), duration (*N*_*t*_) and timestep (*δt*) for different values of the control parameters (*m*_1_, *k*_*D*_) (see Materials and Methods section ‘Simulation methodology’). We define the [*n*_*c*_×*n*_*g*_] transcriptomic matrix ***G***(*m*_1_, *k*_*D*_) once the system has reached steady state in the simulation. We observed that the steady-state distributions for individual genes (e.g. *g*_1_, shown in Fig. 2B) shift their mean as the control parameter, *m*_1_, is varied and exhibit bimodality at the bifurcation point, *m*_1_=*m*_1*c*_=3, as expected for saddle-node bifurcations.

Having verified that our model simulates a system that undergoes a high dimensional saddle-node bifurcation driven by a two-gene driver core, we used it to examine the effects of noise and a large number of responding genes on the theoretical predictions (Eqns 2-4). As predicted, we found that *ω*_1_(*m*_1_), the largest eigenvalue of the covariance of ***G***(*m*_1_), is maximal at the critical value *m*_1*c*_ (darker line in Fig. 2D), and the increase is significantly larger than can be obtained from a null distribution (lighter line in Fig. 2D) that lacks the correlations between genes of the model (see Appendix S1, section 4). This contrast between the data and the null can be understood by considering the bimodality of the transcriptomic distribution at the bifurcation. Far from the bifurcation, the transcriptomic distribution is unimodal, and all *ω*_*i*_ values scale with the noise scale *s*, which is undirected and therefore unaffected by resampling, yielding 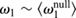 (Fig. S3, left and right panels). However, at the saddle-node bifurcation, the transcriptomic distribution is bimodal, so *ω*_1_ scales with the distance between the two modes (Fig. S3, center top); marginal resampling of transcriptomes at the bifurcation yields new modes and the increased dimensionality of the bifurcation diminishes 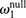, compared with *ω*_1_ (Fig. S3, center bottom). While Fig. S3 only demonstrates the bifurcation bimodality in *g*_1,2_, the full transcriptomic bimodality can be visualized by computing 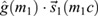, the normalized projection of the transcriptome of each cell along the principal covariance eigenvector. The distribution of this projection is densely centered around different fixed points to the right and left of *m*_1*c*_, but widens significantly at *m*_1*c*_ as there is non-zero probability for both transcriptomic modes (Fig. 2E).

Although this toy model has an explicit bifurcation parameter, often the controls for specific developmental transitions are unknown in scRNA-seq data and the developmental time for each cell is inferred (pseudotime). To verify that similar spikes are expected even when the covariance is measured as a function of an indirect measure of a control parameter, such as pseudotime, we performed a pseudotime analysis on our toy model (Fig. S4A). In particular, we reduced the dimensionality of ***G*** using the SPRING method (Weinreb et al., 2018a), in which the (*x*, *y*) coordinates of each cell are determined by optimally placing each cell closest to its 4 nearest neighbors in the space of the top 10 gene-wise principal components (PC) of highly variable genes (Weinreb et al., 2018a), and computed their pseudotime using Slingshot (Street et al., 2018), in which pseudotime is approximated by the distance of the reduced-dimension data to a spline fit from one end of the data to the other (see Appendix S1, section 7.2 for details). We then binned the replicates (cells) by their pseudotime rank in 100 cell bins, and computed the principal covariance eigenvalue in each bin. We found that *ω*_1_ exhibited a statistically significant spike precisely where the distance between the average control parameter in the pseudotime bin was closest to the critical parameter 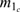
(Fig. S4B). This result suggests that our analytical framework may be directly applicable to detecting similar bifurcations in pseudotime-sorted scRNA-seq data.

Because, in this example, we have an explicit generative model (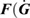), given by Eqns 5 and 6), we can validate that just as *m*_1*c*_ resembles a bifurcation from analysis of the covariance matrix, it also resembles a bifurcation of the full noisy GRN, from analysis of the Jacobian. We show that the maximum negative eigenvalue (*λ*_*d*_) of the Jacobian (
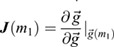
) for this network approaches 0 from below as *m*_1_→*m*_1*c*_ (Fig. 2F). We also show that at *m*_1*c*_, the direction of maximal covariance, is given by the corresponding eigenvector of the Jacobian (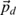), as the Euclidean distance between 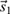, the principal eigenvector of the covariance, and 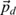 approaches 0, as *m*_1_→*m*_1*c*_ (Fig. 2F). Thus, although the finite system size (*n*_*c*_) prevents, or regularizes, *ω*_1_ from diverging, and 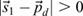, *ω*_1_ is still at its largest and the eigenvectors are in closest correspondence at the bifurcation.

To empirically benchmark the principal covariance eigenvalue as a bifurcation indicator, we selected genes with strong connections (|*α*_*i*_−0.5|>0.25) and computed the DNB order parameter as a function of *m*_1_ (see Appendix S1, section 2) (Liu et al., 2012; Chen et al., 2012; Li et al., 2019; Chen et al., 2018). We found that the variation of *ω*_1_ matched the variation of the DNB order parameter (Fig. 2D), providing empirical support for using *ω*_1_ to identify bifurcations. Importantly, computing *ω*_1_ did not require preprocessing, while computing the DNB order parameter requires a preprocessing step to select the DNB genes (Chen et al., 2018). Additionally, *ω*_1_ is computationally more efficient, as it is obtained via the singular value decomposition of the gene expression matrix, whereas the DNB order parameter is obtained via the correlation matrix across tens of thousands of genes (Hastie et al., 2009).

Although the covariance eigen-decomposition provides insight into the timing and direction of a bifurcation, Eqn. 4 predicts that the (Pearson) correlation coefficients between genes may help determine which genetic relationships are most critical for the dynamics at the bifurcation. We found that, for low and high *m*_1_, when the network only has one fixed point, the distribution of correlation coefficients *R*_*ij*_ is strongly centered around 0 (Fig. 2G). However, at the bifurcation, this distribution spreads out to ± 1, as predicted in Eqn. 4 (Fig. 2G). To determine whether the gene pairs that yielded large *R*_*ij*_ corresponded with critical gene relationships in our network, we plotted *R*_*i*,*d*(*i*)_: the correlation between all responder genes and their drivers, sorted by their connection strength *α*_*i*_. We found that these correlation coefficients were much more strongly indicative of the responder-driver dependency (*α*_*i*_) at bifurcation (Fig. 2H, green) rather than far away from the bifurcation (Fig. 2H, red and blue). Again, the GRN model makes it explicit that, although the correspondence between geometry and dynamics is not universal, owing to the high-dimensionality of the system, in the vicinity of a bifurcation, a form of dimensionality reduction emerges that enables the gleaning of geometric characteristics directly from the dynamics. Thus, entries of a correlation matrix with high magnitude at a bifurcation may be reliable indicators of mechanistic gene-regulatory features.

We further used our GRN model to probe a pitchfork bifurcation induced by varying *k*_*D*_ (Fig. S5A). Unlike the example of a saddle-node bifurcation, we observed that *ω*_1_ does not peak at the bifurcation parameter *k*_*Dc*_=0.5, but rather begins to increase (Fig. S5B). This feature directly follows our interpretation that *ω*_1_ corresponds to the distance between the two modes of transcriptomic distribution. Whereas the bimodality of the saddle-node bifurcation results from the discontinuous transition between states, the bimodality of pitchfork bifurcation emerges continuously from its root and becomes more pronounced as the control parameter is increased. Therefore, the distance between the modes (*ω*_1_) increases with the control parameter. By clustering the cells according to their transcriptomic mode, or branches, we are able to recover the bifurcation signature predicted by Eqn. 2 (Fig. S5C), but we note that precise clustering requires prior knowledge (e.g. how many clusters there are).

As developmental decisions are often modeled as noise-induced state transitions between co-existing transcriptomic states (Weinreb et al., 2018b; Zhou et al., 2021), rather than bifurcations, we sought to determine whether our framework could distinguish these two possibilities. We used our model gene network (Fig. 2A) to explore the noise-induced transition possibility by varying the noise scale *s* of the network at fixed values of the bifurcation parameters (see Appendix S1, section 5 for details). We found that the principal covariance eigenvalue exhibited unique step-like dynamics as the stochasticity was varied (Fig. S6), which was unlike either the one-to-one (Fig. 2D) or one-to-many (Fig. S5B) bifurcation examples explored, demonstrating that analysis of the covariance dynamics in transcriptomics should enable distinguishing between noise-induced transitions and bifurcations.

In this example, we have demonstrated the applicability and power of the theoretical infrastructure outlined above to analyze a high-dimensional and noisy dynamical system undergoing a variety of bifurcations, by uncovering its crucial aspects, including its location, direction in gene space and influential genetic relationships. These calculations are also computationally simple; although covariance matrices can be cumbersome to compute for large numbers of genes and cells, reduced singular value decomposition can be used to determine quickly its largest eigenvalue and eigenvector, which is all our approach requires. Notably, our results apply only if the system is measured at steady state, otherwise there is no reason to anticipate clear divergences in the distribution of eigenvalues, transient bimodality or equivalence between the covariance and Jacobian principal directions (Fig. S7B-D).

### Covariance analysis pinpoints a bifurcation in neutrophil development

**Fig. 3.**
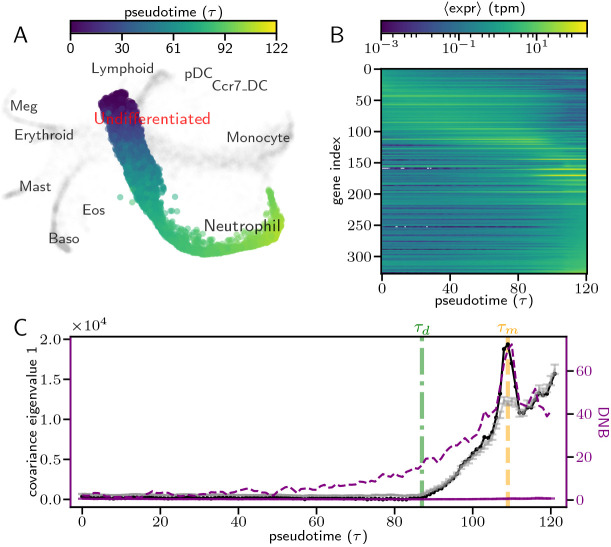
**Covariance analysis of temporal scRNA-seq data shows signatures of developmental bifurcations.** (A) SPRING visualization for each cell (point) in an *in vitro* scRNA-seq experiment of mouse hematopoeitic stem cell differentiation (Weinreb et al., 2020). Cells in the pseudotime trajectory analyzed are colored accordingly (blue to yellow), whereas others are gray. SPRING coordinates, cluster labels, pseudotimes and a similar visualization were first reported by Weinreb et al. (2020). (B,C) Observations of neutrophil trajectory as a function of pseudotime calculated for each of 121 bins of pseudotemporally adjacent cells. All bins had 1000 cells except for the last one, which had 1310 cells, and had a 50***%*** overlap with neighboring bins. (B) Average gene expression in pseudotemporal bins for highly expressed [max (〈expr〉)>1] and highly varying [coeff. of var.(〈expr〉)>0.5] genes. (C) Principal covariance eigenvalue (black) compared with a statistical null (gray, details in Appendix S1, section 4), shifted to have 0 min. Error bars of null are ±1s.d. DNB order parameter also shown, where the DNB comprises neutrophil marker genes (purple dashed line) or is the average of many random gene sets (purple solid line, see Fig. S9 for details). Green and yellow dashed lines indicate the developmental transition times *τ_d_* and *τ_m_*.

Having verified that gene-gene covariance can be used to provide insight into transitions in a simulated genetic context, we applied our analysis framework to a recently published scRNA-seq data set of mouse hematopoietic stem cell (HSC) differentiation (Weinreb et al., 2020). In this experiment, HSCs were isolated *in vitro*, barcoded, plated in a media that supports multilineage differentiation (day 0) and subsequently sampled for single-cell sequencing using inDrops (Klein et al., 2015 ; Plasschaert et al., 2018) on days 2, 4 and 6. The resultant transcriptomic matrix (25,289 genes in 130,887 individual cells) was visualized in 2*D* using the SPRING method (Weinreb et al., 2018a) (Fig. 3A), using 4 nearest neighbors in the top 50 PC space for highly variable, non-cell cycle genes (Weinreb et al., 2018a). Each cell was then associated with one of 11 different cell types (annotations in Fig. 3A) based on its position in the SPRING plot and expression of cell type-specific marker genes (Weinreb et al., 2020). Cells that belonged to the developmental transition from multipotent progenitor (MPP) to neutrophil were identified by recategorizing cells as a cell-label distribution, and ranking cells by their similarity to fully committed neutrophils (see details in Appendix S1, section 7.1). The 61,310 cells identified as belonging to the neutrophil transition were sorted into a neutrophil pseudotime trajectory (Fig. 3A) by ranking cells according to their similarity with the earliest pluripotent cells (see Appendix S1, section 7.1). This data-specific pseudotime algorithm was validated via the clonal barcodes of the cell, by ensuring that the MPP cells in the trajectory included neutrophil clones, and via the sequencing time, by ensuring that cells collected earlier were ranked earlier in the trajectory. Thus, several features of this trajectory make it ideal for applying our analysis framework: it includes a large number of cells, enabling statistically reliable covariance measurements; it is robust to the systematic, temporal controls of sequencing -time and cellular barcodes; and it is part of hematopoiesis, a well-characterized developmental process that enables the comparison of our findings with past work. We found that this trajectory was extremely dynamic, as the expression of hundreds of highly expressed genes is temporally variable, with large groups of genes either monotonically increasing or decreasing (Fig. 3B).

To determine whether the transitions from HSC to neutrophil were due to bifurcations in transcriptomic space, we split the neutrophil trajectory into overlapping bins of 1000 cells (last bin had 1310) and applied our covariance analysis to the full, row (cell)-normalized transcriptomic matrix at each bin ***G***(*τ*). We found that the largest eigenvalue of the covariance of the full gene expression matrix [*ω*_1_(*τ*), dark line in Fig. 3C] exhibited very little variation for *τ*<*τ*_*d*_=85, but began to increase at *τ*_*d*_ and exhibited a significant spike at *τ*_*m*_=109, which is indicative of a bifurcation. To determine whether *ω*_1_ changes were statistically significant, we computed the corresponding statistical null (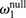, lighter line in Fig. 3C; details in Appendix S1, section 4) and found that the large peak at *τ*_*m*_ was easily distinguishable from the null. To benchmark our result against established bifurcation identification methodologies, we computed the DNB order parameter (Chen et al., 2012) across pseudotime, using known marker genes for neutrophils and neutrophil progenitors (Weinreb et al., 2020) as the DNB (see details in Appendix S1, section 2). We found that the DNB order parameter (Fig. 3C, purple dashed line) exhibited similar dynamics to *ω*_1_(*τ*), but we emphasize that, unlike the computation of *ω*_1_(*τ*), computing the DNB requires gene filtering, as random sets of genes did not exhibit bifurcation signatures (Fig. 3C, purple solid line; Fig. S9). We also verified that bin size did not generally impact the dynamics of *ω*_1_(*τ*) (Fig. S10).

We first focus our attention at the dynamics at *τ*_*m*_, after which we will address those observed at *τ*_*d*_. As this pattern of a statistically significant spike following near-constant *ω*_1_ echoed the observed behavior of a saddle-node bifurcation in our toy model (Fig. 2D), we speculated that at *τ*_*m*_ there was a one-to-one transcriptomic state transition. Additionally, to verify that the temporal trend in Fig. 2D was not limited to the diffusion-based pseudotime algorithm used by Weinreb et al. (2018a), we recalculated pseudotime using Slingshot (Street et al., 2018), and found the same rise and peak of *ω*_1_ (see Appendix S1, section 7.2 and Fig. S11A,B).

We focus now on our observations in proximity to *τ*_*d*_. As the increase in *ω*_1_ at *τ*_*d*_ strongly resembled the pitchfork bifurcation of our toy model (Fig. S5B), as well as the proliferation of cell fates seen in high-resolution time-course scRNA-seq experiments (Nitzan and Brenner, 2021), we hypothesized that the increase of *ω*_1_ at *τ*_*d*_ was also due to transcriptomic state changes. Further evidence of a developmental transition is that, at *τ*_*d*_, the distribution of expression of each gene across cells begins to significantly shift toward higher values (Fig. S10C). However, the precise nature of this developmental transition is unclear, because, in contrast to our toy model, 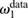 is nearly indistinguishable from 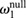 during the increase.

**Fig. 4.**
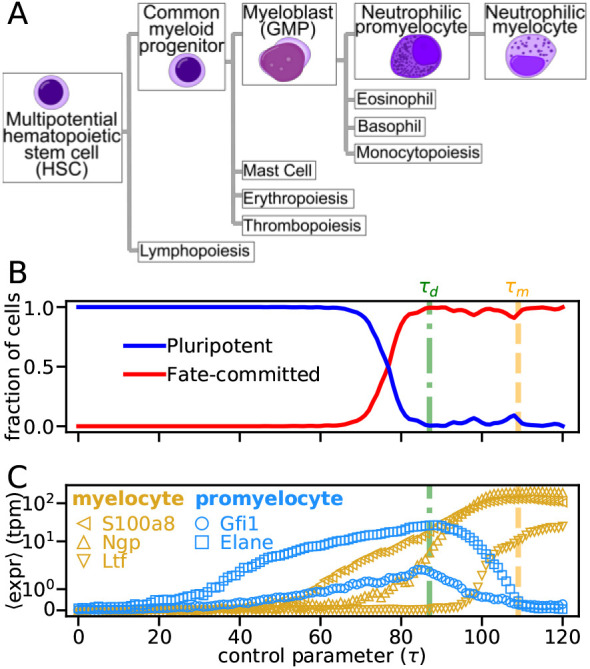
**Detected bifurcations correspond to biologically characterized developmental transitions.** (A) Schematic of neutrophil development, beginning from hematopoietic stem cells and ending at the neutrophil myelocyte – a committed neutrophil progenitor. Lines indicate naturally occurring progeny, other than the cell type itself. Subsequent neutrophil-committed fates (neutrophil metamyelocyte, band cells and neutrophils) are not shown. Cell type images by A. Rad and M. Häggström. CC-BY-SA 3.0 license. (B) Fraction of cells in each cell type, based on annotated clustering in Weinreb et al. (2020). (C) Average expression of promyelocyte (blue) and myelocyte (gold) marker genes (Weinreb et al., 2020). Error bars (s.e.m.) are smaller than symbols.

To determine whether the transcriptomic state transitions we identified had biological significance, we compared our findings against the tree of cell fates for neutrophil development (Fig. 4A). We found (Fig. 4B) that *τ*_*d*_, the moment *ω*_1_ begins to increase, corresponded well with the moment in pseudotime that cells switch between the endpoints of this tree: from not expressing any terminal-fate marker genes to primarily expressing neutrophil cell-fate markers (Weinreb et al., 2020). At a more granular developmental level, the pseudotimes highlighted by our covariance analysis align with specific transitions between intermediate neutrophil progenitor states (Fig. 4A). These transitions include: (1) one-to-many cell fate changes (i.e. decisions), such as the transition between a granulocyte monocyte progenitor (GMP, or myeloblast) and any of its four terminal fates (neutrophil, monocyte, eosonophil and basophil); and (2) one-to-one cell fate changes (i.e. maturation), such as the transition between promyelocyte and myelocyte (Weinreb et al., 2020; Borregaard, 2010; Ostuni et al., 2016). *τ*_*d*_ corresponds well with the pseudotime at which promeylocyte marker genes are maximal (Fig. 4C and Fig. S11C), suggesting a connection between *τ*_*d*_ and the one-to-many change from GMP to promyelocyte. This can perhaps be understood in light of other one-to-many state transitions, such as a pitchfork bifurcation (Fig. S5A), where *ω*_1_ increases steadily if different branches are left unclustered (Fig. S5B). Although the null and signal were significantly closer in the neutrophil trajectory than in the toy model, this discrepancy may be related to the difficulty in identifying one-to-many transcriptomic changes in non-ideal conditions, compared with one-to-one transitions (see Appendix S1, section 6 and Fig. S8 for details). Accordingly, the increase of *ω*_1_ at *τ*_*d*_ suggests that, although cells in the neutrophil trajectory are expected to include only the neutrophil lineage branch of GMP, they may in fact include other GMP lineages, such as eosonophils or basophils.

Conversely, *τ*_*m*_ corresponds well with the pseudotime when myelocyte marker genes are maximal and promyelocyte genes have reduced expression (Fig. 4C and Fig. S11C), suggesting that *τ*_*m*_ indicates the transition point between these two cell fates. Although the myelocyte marker genes begin increasing earlier than *τ*_*m*_ in the trajectory, this may be because of additional cellular processes that smooth out their dynamics over the course of a cell-fate transition. Alternatively, the marker gene dynamics may indicate that the transition at *τ*_*d*_ is connected to the transition at *τ*_*m*_; e.g. the eigenvalue dynamics at *τ*_*d*_ may hint at an early bias toward the ultimate myelocyte transition, similar to other developmental biases that have recently been identified in hematopoiesis (Wang et al., 2022).

Thus, by using Eqn 2 to quantify the geometry of neutrophil development, we were able to recover the known GMP-neutrophil cell fate decision, qualify the trajectory as likely including other lineages and pinpoint a maturation step in neutrophil development. Importantly, this analysis also highlights the difficulties in using the principal covariance eigenvalue alone to characterize bifurcations, as one-to-many bifurcations in particular may be extremely sensitive to small errors or biases. To address these difficulties, and provide additional insights into possible dynamical transitions, we now leverage a key feature of the covariance analysis outlined above: that signatures of the underlying mechanisms driving a system through a bifurcation are evident in its principal covariance eigenvector.

### Covariance eigenvectors provide interpretable low dimensional representations of neutrophil bifurcations

Perhaps the most surprising consequence of the Continuous Time Lyapunov equation, encapsulated in Eqn. 3, is that a high-dimensional bifurcation eigenvector, which is a characteristic of the underlying Jacobian of the system, is directly calculable from the transcriptomic-state data, as it equals, up to a sign, the principal covariance eigenvector 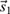. This result motivated us to probe 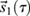, the principal eigenvectors of the covariance matrix as a function of pseudotime, and in particular its structure in the vicinity of *τ*_*d*_ and *τ*_*m*_, to glean further insight into the biological nature of our detected transition points.

**Fig. 5.**
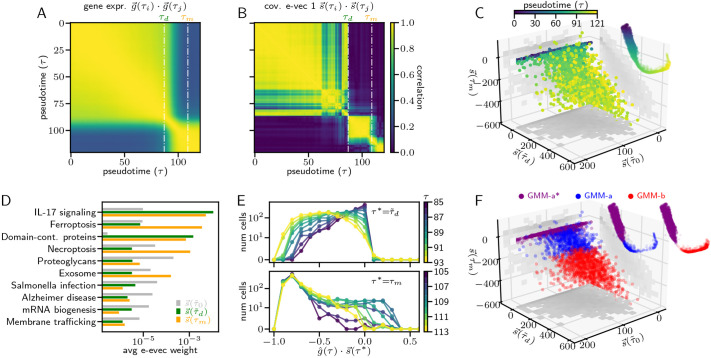
**Analysis of high-dimensional bifurcation directions.** (A) Pairwise correlation of average normalized gene expression in each pseudotime bin. (B) Pairwise correlation of the principal covariance eigenvector of each pseudotime bin. (C) Projection of normalized gene expression along principal eigenvectors at *τ*_0_, *τ*_***d***_ and *τ*_***m***_. Each dot is a cell and its color indicates the pseudotime. Inset shows corresponding position of the cell in the SPRING plot (Fig. 4A). (D) Average weight, per gene, of the highest weighted categories in the KEGG database for *Mus musculus*, for the principal covariance eigenvectors at *τ*_0_,*τ*_***d***_, and *τ*_***m***_. (E) Distribution of gene expression projected onto 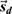, near *τ*_***d***_ (top), and distribution of gene expression projected onto 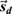 near *τ*_***m***_ (bottom). (F) As in C, but the color of points (cells) indicates their cluster in the GMM; insets are split by cluster.

We first sought to determine the uniqueness of 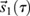, as it is extremely high dimensional, by measuring how it varies across pseudotime, compared with average gene expression. We found that the correlation of average gene expression across pseudotime exhibits an approximate two-block structure throughout the trajectory, in which expression is well correlated in *τ*∈[0, 80] and again in *τ*∈[90, 121*t*] (Fig. 5A), hinting at the existence of two gene expression states. Interestingly, the correlation of 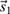 was significantly more detailed, exhibiting as many as six distinct blocks, with higher positive correlation and lower negative correlation than seen in expression (Fig. 5B). Importantly, *τ*_*d*_ and *τ*_*m*_ align well with transitions between blocks, further bolstering their significance as markers of developmental transitions. Thus, the variation of 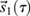, even when it is high dimensional, may reveal significant detail in the structure of a developmental trajectory.

We next sought to examine whether 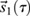 contain axes of a simplified space along which to examine the trajectory. As the correlation between eigenvectors exhibited distinct blocks, two of which had bifurcative dynamics, we used 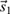 near the beginning of the trajectory and near *τ*_*d*_ and *τ*_*m*_ to revisualize the neutrophil trajectory. For *τ*_0_ and *τ*_*d*_, we used an 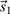 toward the middle of its block (at 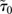 and 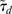, respectively; see Appendix S1, section 8 for details) to ensure that the direction had stabilized; however, for *τ*_*m*_ we used the vector precisely at *τ*_*m*_, as the direction of a one-to-one transition may only be observable at the moment of bifurcation (Eqn. 3). We found that for the majority of the trajectory, the variation was localized to 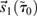, but starting near *τ*_*d*_, cells began to vary along both 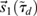 and 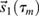 (Fig. 5C). As the variation along 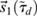 and 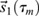 coincided, and they had a high correlation of 0.67 (Fig. 5C), while both being nearly completely orthogonal to 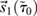, we sought to determine their differences. We found that whereas the fraction of variation of gene expression along 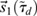 begins to increase near *τ*_*d*_ and remains high even around *τ*_*m*_ (Fig. S12A), highlighting the importance of 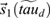 for the transition at *τ*_*m*_, 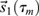 accounts for only a significant fraction of variation very close to *τ*_*m*_ (Fig. S12A), suggesting additional features that are distinct from 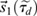 (Fig. S12A). Additionally, the projection of the vector tangent to gene expression onto these eigenvectors appears high for both bifurcation eigenvectors after *τ*_*d*_, suggesting that they are simultaneously involved in the gene expression dynamics (Fig. S12B) and that, at least from *τ*_*d*_ onward, the trajectory appears multi-dimensional. We found hints of the distinctly 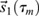 features in the gene expression projection (Fig. 5C), where towards the end of the trajectory, when cells have already deviated far from the 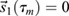 plane, some of the cells appear closer to that plane. Thus, the increased variance along 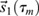 reflects the spike of *ω*_1_ at *τ*_*m*_ (Fig. 4C), and further illuminates it by showing that the direction of the bifurcation is toward the pluripotent state.

As these directions in transcriptomic space revealed new dynamics, we probed the loadings of each eigenvector to determine its functional relevance. To do so, we computed the average weight of each functional category in the KEGG database for *Mus musculus* in the eigenvector (Kanehisa, 2019), i.e.
(7)

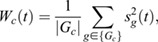
where *W*_*c*_(*t*) is the average weight of category *c* at pseudotime *t*, *G*_*c*_ is the set of genes in the transcriptomic matrix that map to category *c*, and *s*_*g*_(*t*) is the weight of gene *g* in the principal covariance eigenvector at pseudotime *t*. We show *W*_*c*_(*t*) for 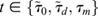 in Fig. 5D for the five categories with highest *W*_*c*_ at each of those pseudotimes. We found that 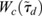 and *W*_*c*_(*τ*_*m*_) were heavily weighted for interleukin 17 (IL17) signaling, a key pathway for controlling infection (Monin and Gaffen, 2018), which has been shown to be activated by neutrophils (Li et al., 2010), to promote neutrophil recruitment (Cua and Tato, 2010) and to aid in the formation of neutrophil exctracellular traps (Lin et al., 2011). Interestingly, 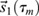 is also highly weighted for regulated cell death mechanisms, including ferroptosis and necroptosis. The high weight for these mechanisms may indicate that the transition at *τ*_*m*_ includes a functional gain, as ferroptosis has recently been demonstrated as a mechanism that neutrophils use to combat glioblastoma cells (Yee et al., 2020) and is also associated with neutrophil extracellular traps (Chen et al., 2021). Alternatively, these mechanisms may have resulted from the activation of cell death within the neutrophil population itself, as necroptosis has been suggested as a population-control mechanism to prevent tissue damage that can occur from an overaccumulation of neutrophils at an infection site (Wang et al., 2018).

Last, we used 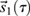 to examine whether the increased *ω*_1_ at *τ*_*d*_ and *τ*_*m*_ is due to the emergence of a second distinct cellular population or, alternatively, to an increase in the transcriptomic variance. Concretely, if the increased *ω*_1_ is due to a second population of cells, then the projection of gene expression at *τ* along 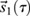 should appear bimodal. We found that this projection widened near both *τ*_*d*_ and *τ*_*m*_ (Fig. 5E and Fig. S12C), reflecting their increased *ω*_1_, and exhibited bimodality at *τ*_*m*_, suggesting the emergence of a second population of cells. This bimodality is also apparent from the gene expression distributions for many of the genes with highest weights in 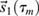, including Fth1, Psap and Ccl6 (Fig. S13). To further disentangle the two populations of cells, we fit a two-peak Gaussian Mixture Model (GMM) to ***G***(*τ*_*m*_) (see Appendix S1, section 9 for details) (Pedregosa et al., 2011). The GMM clearly distinguished the two modes in bimodal gene expression distributions, and showed that many other genes, such as S100a9 and Ngp, which appear initially to have unimodal distributions, also exhibit bimodal structure (Fig. S13).

To evaluate the dynamics of the two cellular populations, we used the GMM to predict the cluster label for all cells in the full trajectory. We found that, for *τ*<*τ*_*d*_, all cells belonged to the same cluster (GMM-a), and began to separate into two clusters (GMM-a and GMM-b) near *τ*_*d*_ (Fig. S14). Additionally, the two clusters exhibit contrasting *ω*_1_ dynamics: GMM-a has increasing *ω*_1_, while GMM-b has a spike in *ω*_1_ at *τ*_*m*_ (Fig. S14). Importantly, the clustering split the cells along 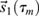, such that cells that were late in pseudotime, but close to the 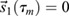 plane, are part of cluster GMM-a, indicating that they are functionally earlier in the neutrophil specification path (Fig. 5F).

Summarizing, we found that the geometry of the data in transcriptomic space, explicated by the principal covariance eigenvector, yielded significant insight into developmental specification dynamics throughout pseudotime, especially at bifurcations. Throughout pseudotime, correlations between eigenvectors were able to pinpoint pivotal trajectory moments, including, and in addition to, those indicated by the principal covariance eigenvalue. Although the eigenvalue analysis alone did not clearly distinguish between the two transitions, the eigenvector analysis highlighted unique mechanistic features at each transition, suggesting they represent distinct developmental events. Eigenvectors at the bifurcations were also helpful in visualizing the trajectory, and inferring the molecular and cellular processes that reshape the developmental landscape. Furthermore, by viewing the bifurcating data along its eigenvector, we were able to discern bimodality and to distinguish between multiple cell fates within the same lineage. Thus, eigenvector analysis appears to be a promising new direction for analysis of pseudotime trajectories.

## DISCUSSION

A singular challenge in understanding cellular fate transitions using transcriptomics has been dimensionality: cell fates are a low-dimensional functional description, a valley in Waddington's landscape, whereas gene-expression profiles are points in a myriad-dimensional space – how can gene expression possibly show the geometry of development? In this study, we have leveraged the continuous-time Lyapunov equation to show that the dynamics of state-variable covariance, even at high dimensionality, are sufficient to assess a crucial aspect of developmental geometry: when and how linear stability is lost to yield a bifurcation. Our central and novel result, based on a restricted region of the transcriptomic trajectories present during the process of hematopoiesis, is that the requisite statistical signatures of a bifurcation are detectable and present during development, even through the complex, high-dimensional lens of sequencing. Although biases and imperfections in data may confound developmental bifurcations, pairing analysis of both the principal covariance eigenvalue and eigenvectors enable us to disentangle multiple transition points, and elucidate mechanistic features that are normally completely hidden in the absence of a candidate mathematical model. Thus, our results have important consequences for the theoretical understanding of developmental transitions, the specific biology of neutrophil development and the analysis of dynamic biological data.

Our finding that a transcriptomic trajectory can have distinct geometric signatures, including durations during which the principal covariance eigenvalue is constant or spikes, has considerable consequences for the theoretical understanding of developmental dynamics. That we saw any consistent behavior in the principal covariance eigenvalue lends significant support to our initial hypothesis that cell fate modifiers operate at a much slower rate than transcriptomic modifiers, because if these occurred on similar timescales, no such statistical signature would be evident, let alone those that align well with current understanding. Additionally, whereas previous statistical analyses of scRNA-seq data found that developmental trajectories appear as monotonic proliferations of cell fates (Nitzan and Brenner, 2021), our focus on a single developmental trajectory enables the distinction of multiple developmental epochs, including durations of development during which cell fates do not undergo qualitative changes, but proliferate (the GMP-to-promyelocyte transition) and change state (the promyelocyte-to-myelocyte transition). Finally, our evidence of bifurcations starkly contrasts with scRNA-seq visualizations that show gene expression varying smoothly along a developmental path, and underscores the importance of understanding both noise and non-linear dependencies when using transcriptomic profiles to classify the fate of a cell (Moris et al., 2016).

Our analysis of the data of Weinreb et al. (2020) also yielded intriguing implications regarding the specific geometry of neutrophil development in mice. In particular, some of the known cell fate changes in neutrophil development were not distinguishable in the covariance eigenvalue trajectory [e.g. from common myeloid progenitors (CMPs) to GMP], which indicates that these changes are less bifurcative than the GMP-to-promyelocyte or promyelocyte-to-myelocyte transitions. This could mean, for example, that even when CMPs differentiate to GMPs, the transition lacks commitment and is dependent on a sustained developmental signal, whereas once cells transition from GMP to promyelocyte, they are committed to becoming neutrophils regardless of an external signal. Alternatively, these non-bifurcative transitions may be driven by early fate biases (Wang et al., 2022), and further research may be necessary to robustly determine which progenitor cell fates are statistically stable. Additionally, in comparing the principal covariance eigenvectors throughout pseudotime (Fig. 5B), it became apparent that the direction along which that fate change happened was well aligned with the direction of the GMP-to-promyelocyte transition. This result may be a sign of distinct, soft directions in transcriptomic space along which cell fates are most likely to change. In addition, as the steady increase and spike in the neutrophil trajectory (Fig. 3C) did not resemble the covariance dynamics of noise-induced state transitions (Fig. S6B), our results suggest that the promyelocyte and myelocyte transitions likely occur due to a loss of stability between fixed points, rather than stochasticity alone.

Aside from these geometric implications, pinpointing bifurcations in pseudotime also enhances analysis of temporal biological data, as it enables the efficient identification of the genes and molecular mechanisms that drive a cell fate transition. At a bifurcation, the principal covariance eigenvectors can aid visualization and highlight critical mechanisms that distinguish clusters (Fig. 5). As the principal covariance eigenvector is equivalent to the Jacobian eigenvector at the bifurcation, and the Jacobian directly reflects gene dynamics, the eigenvector may also be useful for constraining an inferred global Jacobian (Nägele et al., 2014). Additionally, the correlation matrix at a bifurcation may aid in building regulatory network models when combined with previous protein-interaction data or new experimental perturbations (Sun et al., 2015). Furthermore, it may be possible to incorporate our covariance analysis into other indications of pseudotime rank, such as cellular barcodes and low-dimensional distance, to constrain developmental trajectories along bifurcative paths.

Although we focus here on scRNA-seq data, our approach is broadly applicable, and could, in principle, aid in illuminating other aspects of high-dimensional biological dynamics, such as the relationship between development and evolution, or the genomic structural modifications necessary for fate transitions (Buenrostro et al., 2013; Jia et al., 2018). Our analysis was only possible because scRNA-seq experiments can now measure the expression of tens of thousands of genes in hundreds of thousands of cells, enabling accurate covariance measurements. That we found bifurcative events in these data implies that there are low-dimensional, non-linear dynamical systems at play, and that sufficient biological sampling, coupled with physics-based analyses, can reveal the knobs to controllably tilt developmental landscapes.

## MATERIALS AND METHODS

### Continuous time Lyapunov equation for transcriptomic matrices

Let 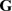 be the steady-state transcriptomic matrix at a single developmental time with *n*_*c*_ rows (cells) and *n*_*g*_ columns (genes), and 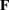 be a set of differential equations describing the molecular interactions that generate 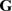, such that
(8)

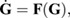
where 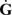 is the derivative of 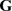 with respect to time. As all cells (columns) in 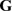 are at steady state at the same developmental time, *τ*, we assume (for the purpose of contradiction) that they are all statistical replicates of the same transcriptomic state, 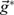, and the full matrix, 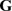, is therefore in the vicinity of the hyperbolic fixed point:
(9)

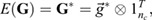
where 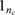 is a vector of *n*_*c*_ values and *E* denotes the expectation operator. The dynamics of 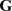 can be by linearized by the distance to the fixed point 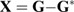, such that
(10)

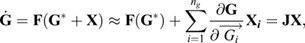
where 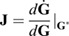 is the Jacobian of 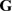 and we have used the fact that, at steady state, 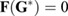.

If 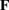 is stochastic and Markovian, then the dynamics of ***X*** can be described as a discretized Ornstein-Uhlenbeck (OU) process:
(11)

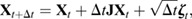
where Δ*t* is the molecular interaction timescale and ***ζ***_*t*;*i*,*j*_ is sampled from *N*(0, *σ*_*i*_), where *σ*_*i*_ is the variance of gene *i*. The gene-gene covariance matrix can then be defined as
(12)

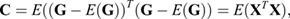
where the superscript *T* denotes transpose and we have approximated 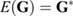. The stationary condition for an OU process (i.e. that 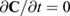) then yields

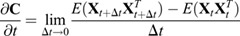

(13)

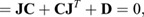
where 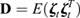; we have used the fact that *E*(***ζ***_*t*_),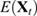,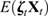 and 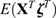 are all 0 (Oku and Aihara, 2018).

### Covariance at bifurcation

If ***J*** is diagonalizable, such that
(14)

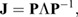
where Λ is a diagonal matrix of eigenvalues (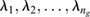) and 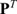 is the square matrix of eigenvectors (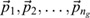), then Eqn 13, often referred to as the continuous-time Lyapunov (CL) equation, can be used to qualitatively assess 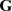. Left multiplying Eqn 13 by 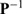 and right multiplying by 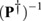, yields
(15)

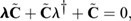
where ^†^ indicates conjugate transpose, 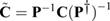 and 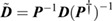. As Λ is diagonal, Eqn 15 can be rewritten elementwise:

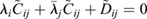

(16)

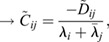
which can be substituted to yield an expression for elements of the covariance
(17)

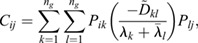
as 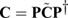. At a bifurcation, max (Λ)=*λ*_*d*_→0, so the *k*=*l*=*d* term in Eqn 17 becomes dominant and
(18)

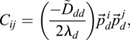
where 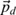 is the *d*^*th*^ column of 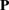.

### Bifurcation eigenvector equivalence

As 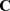 is real and symmetric, the eigenvalue decomposition can be written as a single sum:

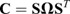

(19)

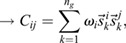
where 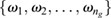 are its eigenvalues, and 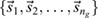 are its eigenvectors, which are normalized to 1. For Eqn 19 to be equivalent to Eqn 18 at a bifurcation, at least one eigenvalue *ω*_*i*_→∞, which we may, without loss of generality, refer to as *ω*_1_. If *ω*_1_≫*ω*_*i*_ for *i*∈[2…*n*_*g*_] then the *k*=1 dominates the sum in Eqn 19, and by equating with Eqn 18 we obtain

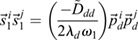


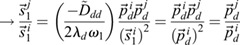


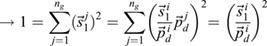


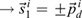


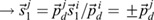

(20)

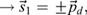
where we have used the fact that 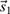 and 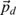 both normalize to 1. Importantly, it is also computationally advantageous to analyze the eigen decomposition of the covariance, rather than the covariance itself, because for large *n*_*g*_, Ω and ***S*** can be obtained directly from the singular value decomposition of ***X***.

### Simulation methodology

To explore our analysis framework on a more biologically relevant gene network (Fig. 2A) we used a Focker-Plank simulation method. For each of the *N*_*c*_=100 cells (*N*_*c*_ chosen by examining how many cells were necessary to accurately detect bifurcations in the neutrophil data (Fig. S10A,B), the expression of gene *i* [*g*_*i*_(*t*;*m*_1_, *m*_2_, *k*_*D*_)] is initialized uniformly randomly in the interval [0,4]. The expression at subsequent time steps [*g*_*i*_(*t*+Δ*t*)] is sampled from a Gaussian distribution *N*(*μ*, *σ*), where
(21)

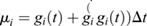

(22)

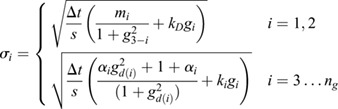
and bounded to be non-negative. The simulations ran for *N*_*t*_=1*e*7 time steps, with Δ*t*=0.01, at a noise scale of 1/*s*=0.05, and the last timestep of each simulation is the steady-state expression ***G***. We verified that *N*_*t*_ was sufficiently large by averaging ***G***(*m*_1_) across cells, and observing that individual genes discontinuously, but predictably, switch their expression at *m*_1*c*_ [Fig. 2C; genes sorted by *d*(*g*_*i*_) and _*i*_] compared with the continuous and unpredictable transitions observed with low *N*_*t*_ (Fig. S7A). In the saddle-node example (Fig. 2), the remaining parameters were *k*_*D*_=1, *m*_2_=3, *m*_1_∈[2, 4], while in the pitchfork example (Fig. S5), *m*_1,2_=1, *k*_*D*_∈[0.24, 5].

### Analysis pipeline

Eqns 2-4 imply an analysis pipeline characterizing bifurcations in high-dimensional temporal data, which we use in this article:

(1) Obtain highly sampled temporal data. Caveat: for data types such as scRNA-seq, where frequent sampling is difficult, and samples may include realizations from many different times, time may be inferable, using, for example, pseudotime inference (see Appendix S1, section 7.1).

(2) Bin the data along the temporal axis.

(3) Compute the largest eigenvalue of the covariance matrix (*ω*_1_) in each bin (e.g. using an off-the-shelf PCA function).

(4) Evaluate whether a bifurcation occurs by comparing *ω*_1_ with a suitable null (see Appendix S1, section 4): spike indicates a one-to-one bifurcation; steady increase indicates a one-to-many bifurcation.

(5) If a bifurcation is detected (e.g. at *τ*_*c*_), compute and examine the principal covariance eigenvector at *τ*_*c*_, as it reflects mechanistic aspects of the underlying dynamical system.

## Supplementary Material

10.1242/develop.201280_sup1 Supplementary information
