# Supplementary material for: A dynamical systems treatment of transcriptomic trajectories in hematopoiesis
Source: Development. 2023 Jun 1;150(11):dev201280. doi: 10.1242/dev.201280 (PMC10281553; doi:10.1242/dev.201280)
Supplement: Supplementary information [file develop-150-201280-s1.pdf]

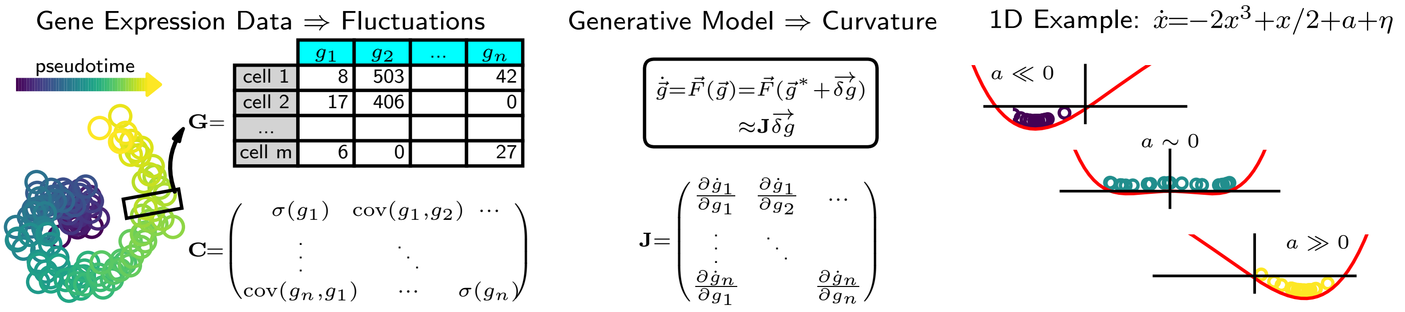

Fig. S1. The relationship between the covariance of a gene expression trajectory and its Jacobian. (A) Schematic of a single-cell RNA-seq dataset arranged by each cell's developmental (pseudo-) time. (Left) Visualization of dataset in two collective gene-expression dimensions. Gene expression matrix at the pseudotime indicated by the rectangle, and its corresponding covariance matrix. (Center) Schematic of a generative model ($\vec{F}$) that could yield the gene expression matrix in (A), and its connection to the Jacobian (**J**). In this model, δg is the deviation of the gene expression vector, $\vec{g}$ from the fixed point, $\vec{g}^*$. (Right) Snapshots of a collection of particles at steady state following the dynamical process defined by $\dot{x} = -2x^3 + x/2 + a$ and uniformly sampled noise for $a = -1$ (left), $a = 0$ (center) and $a = 1$ (right).

Development • Supplementary information

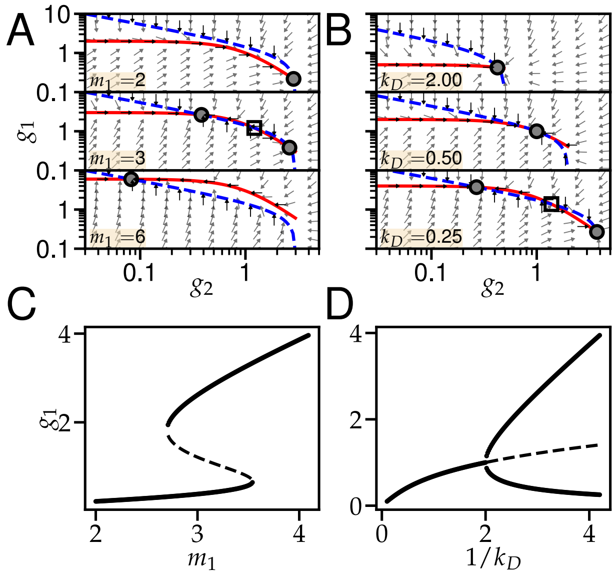

Fig. S2. Analysis of Eqn. 5. (A-B) Phase planes for different parameter sets yield a saddle-node bifurcation (A) or pitchfork bifurcation (B). Solid red line is given by Eqn. S4 while dashed blue line is given by Eqn. S5. Open squares are saddles while closed circles are nodes. Arrow angles are given by $tan^{-1}(\dot{g}_1/\dot{g}_2)$ and are uniform length. (C-D) Solutions to Eqn. S3 while varying m_1 (C) or k_D (D). Solid lines are nodes and dashed lines are saddles. In (A,C) $k_D = 1$, $m_2 = 3$ and in (B,D) $m_{1,2} = 1$.

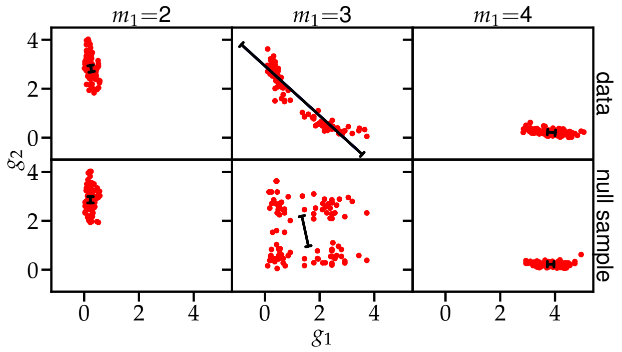

Fig. S3. Effect of resampling on principal eigenvector. Red dots are cells for the data (top) and a single marginal resampling (bottom) before the bifurcation (left), at the bifurcation (center) and after the bifurcation (right). Direction of the black lines corresponds to the principal eigenvector and length corresponds to the principal eigenvalue.

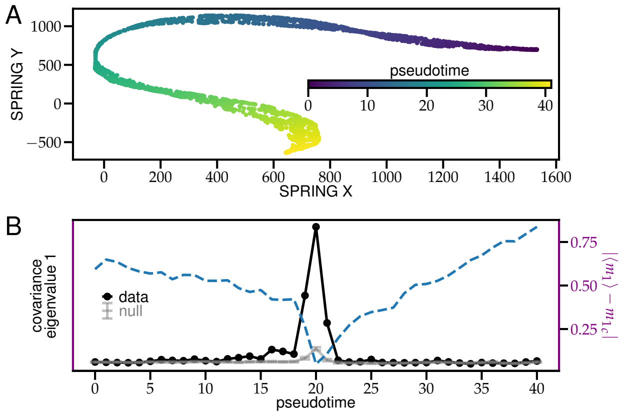

Fig. S4. Pseudotime analysis of saddle-node bifurcation. (A) Representation of simulated cells using SPRING dimen-sionality reduction [1] (min 1 cell, 50 PCs, and 10 nearest neighbors) and Slingshot pseudotime inference (sample size 2000 cells) [2]. (B) Principal covariance eigenvalue (ω_1) plotted as a function of pseudotime (dark dots) as well as the distance of the bifurcation order parameter (m_1 from its critical value (purple dashed line. DNB order parameter as functions of pseudotime. The peak of ω_1 coincides with the minimum distance between m_1 and its critical value.

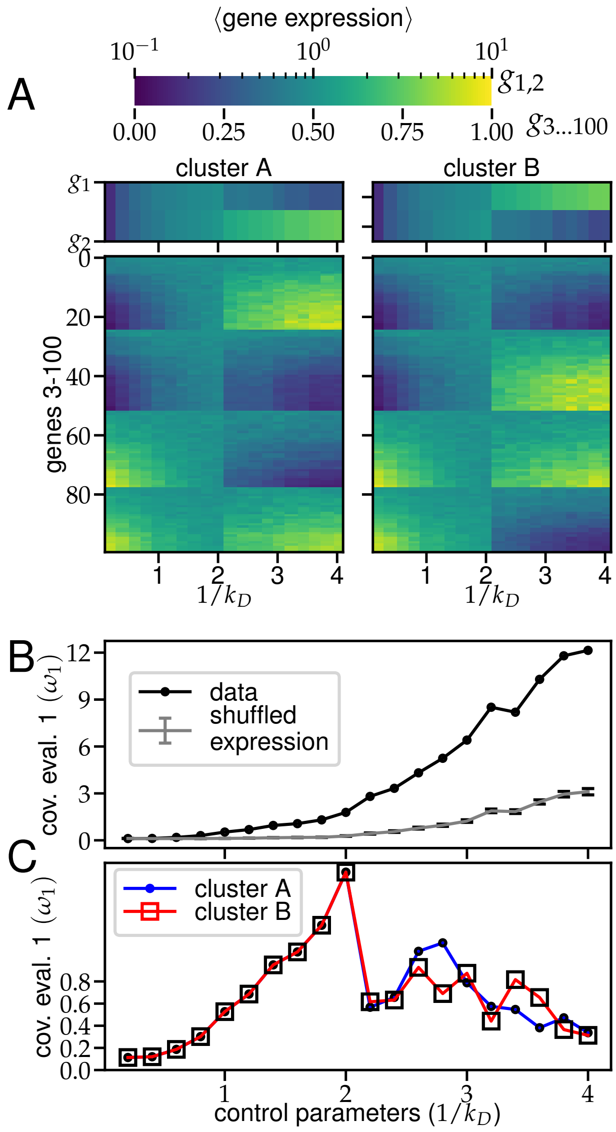

Fig. S5. Pitchfork bifurcation analysis. (A) Gene expression as function of the bifurcation variable τ, separated by cluster.(B) ω_1 and null for unclustered data. (C) ω_1 for clusters.

Development • Supplementary information

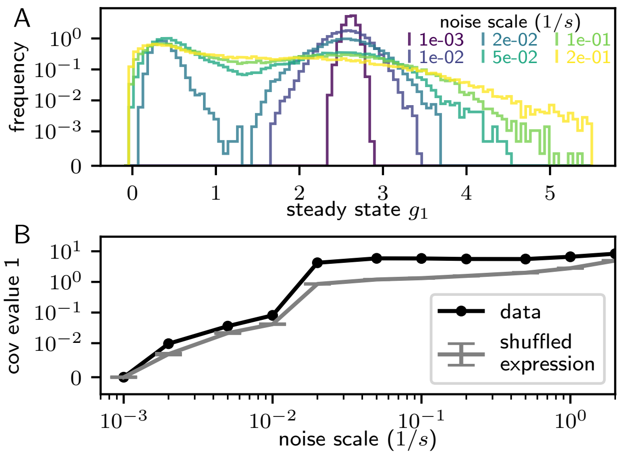

Fig. S6. Covariance eigenvalue signature for a noise induced transition. (A) Steady state distributions for the expression of g_1 at varying noise scales. (B) Principal covariance eigenvalue as a function of noise.

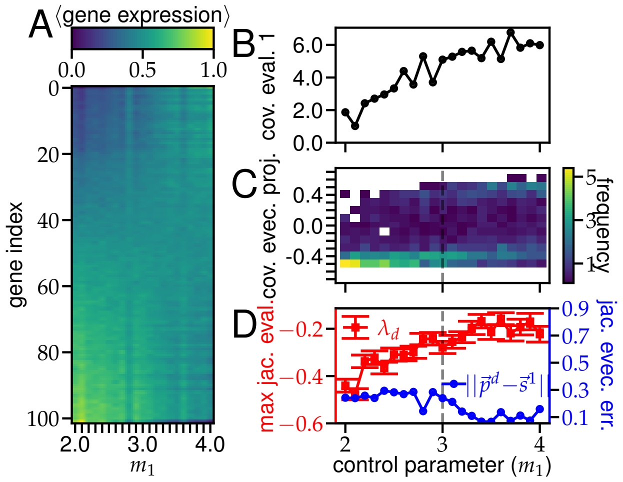

Fig. S7. Unequilibrated saddle-node bifurcation analysis. (A)-(D) Corresponding plots for Fig. 2 (C)-(F), respectively, when the simulation is runs for a $N_t = 500$ iterations rather than $N_t = 50000$.

Development • Supplementary information

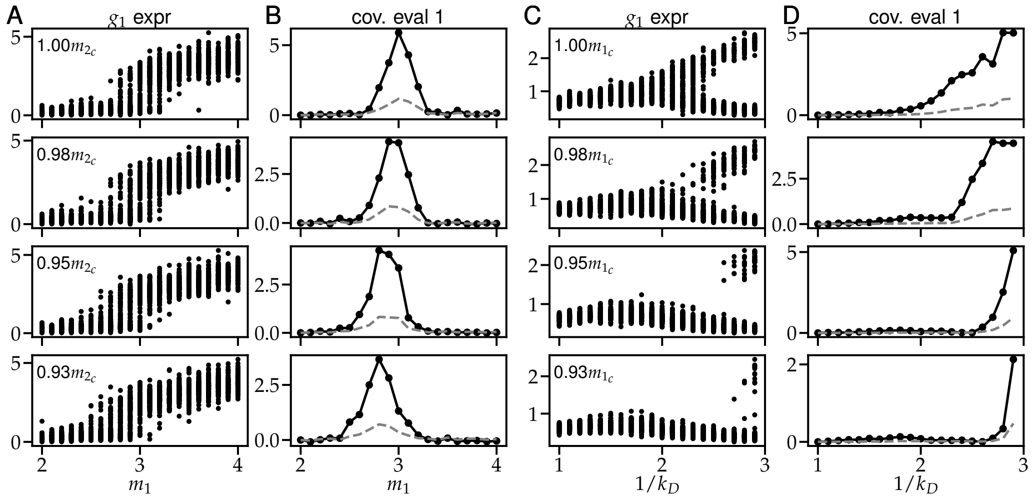

Fig. S8. The effect of small errors in parameter values for saddle node and pitchfork bifurcations. (A) Results of the saddle node bifurcation with 0,2%,5%, and 7% error in the value of the m_2 parameter in Eqn. 5, while m_1 is varied to induce a saddle-node bifurcation. (B) Principal covariance eigenvalue ω_1 and its corresponding null, shifted to have min 0, for the data from part a. (C-D) Same as (A-B), but for errors in the m_1 parameter, while k_D is varied to induce a pitchfork bifurcation.

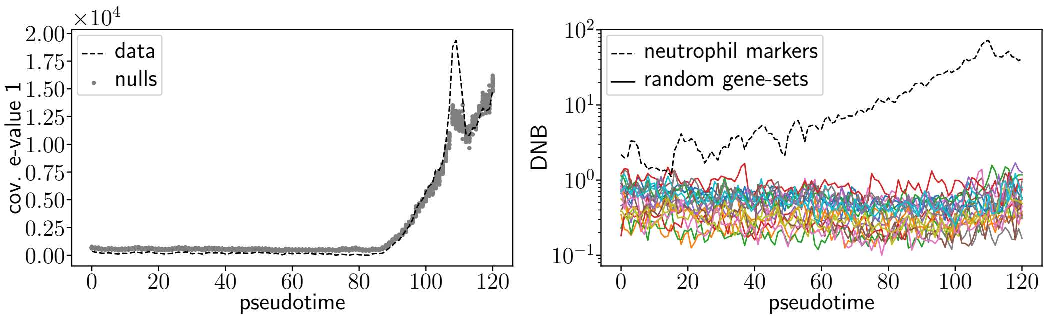

Fig. S9. Statistical nulls for bifurcation order parameters. Left: Principal covariance eigenvalue for the gene expression bins of the neutrophil trajectory (dashed line), and the randomly resampled gene expression bins (gray points). Right: DNB for neutrophil marker genes (dashed line) and for $n = 20$ different randomly selected gene sets. The randomly selected gene sets had the number of genes as the number of neutrophil markers.

Development • Supplementary information

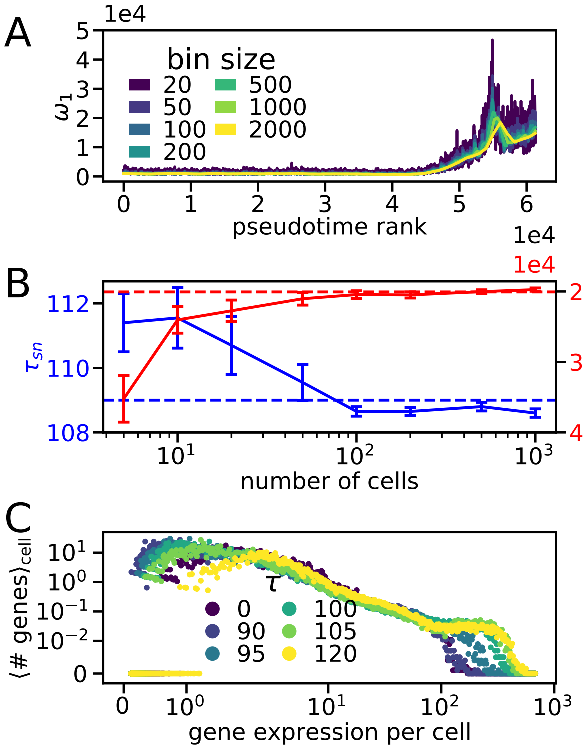

Fig. S10. Distributional properties of the neutrophil pseudotime trajectory. (A) Effect of varying the bin size on principal covariance eigenvalue. (B) Effect of undersampling a bin of 1000 cells on the detected saddle-node bifurcation time and magnitude. (C) Distribution of gene expression during the trajectory.

Development • Supplementary information

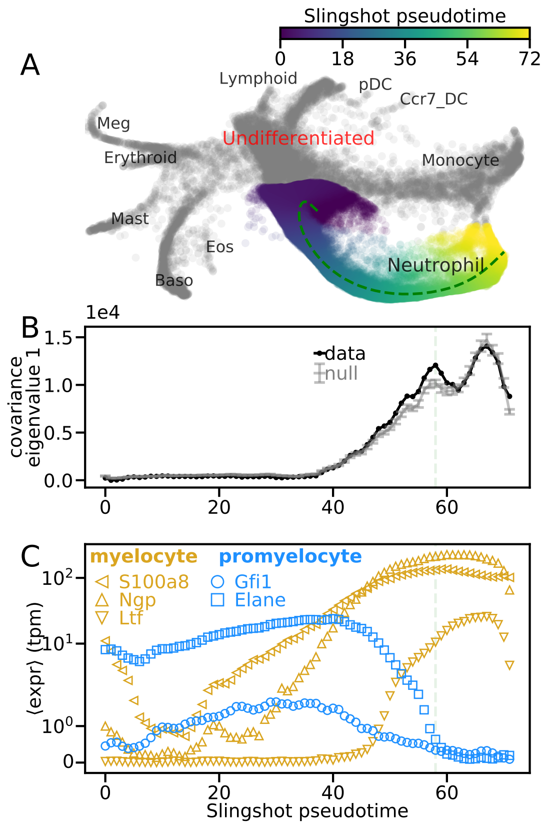

Fig. S11. Bifurcation characterization using Slingshot pseudotime algorithm. (A) Neutrophil development obtained by applying Slingshot to hematopoiesis scRNA-seq data [3]. Principal curves were approximated to 1000 points by setting approx_points = 1000 in the slingshot function as increasing approx_points further did not affect results. (B) Largest covariance eigenvalue (black) compared with a statistical null (gray, details in Section 4) in each 1000 cell pseudotemporal bin, shifted to have 0 min, using the Slingshot pseudotime ordering. Error bars of null are one SD. (C) Average expression of promyelocyte (blue) and myelocyte (gold) marker genes in Slingshot pseudotemporal bins [3]. SEM error bars are smaller than symbols. Light green line in (B-C) indicates peak of bifurcation window.

Development • Supplementary information

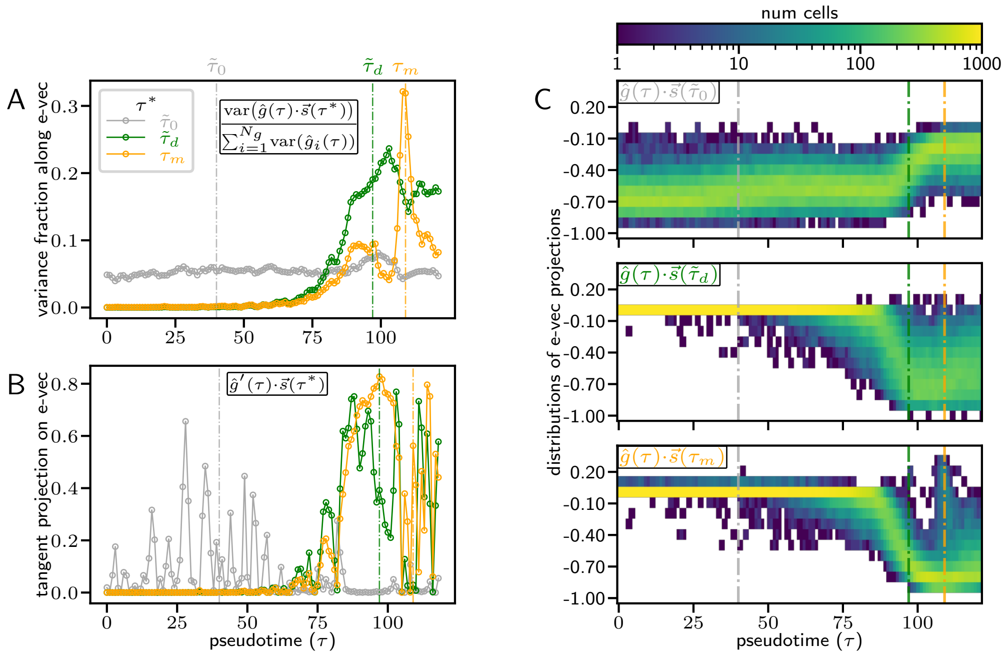

Fig. S12. Projections of gene expression onto bifurcation eigenvectors. (A) Fraction of variance along each of the three eigenvectors, as a function of pseudotime. (B) Mean projection of eigenvectors onto $\hat{g}'(\tau)$, the normalized vector tangent to gene expression (obtained via finite difference). (C) Distribution of normalized gene expression for each cell projected onto the covariance eigenvector at τ_0 (left) τ_d (center) and τ_m (right).

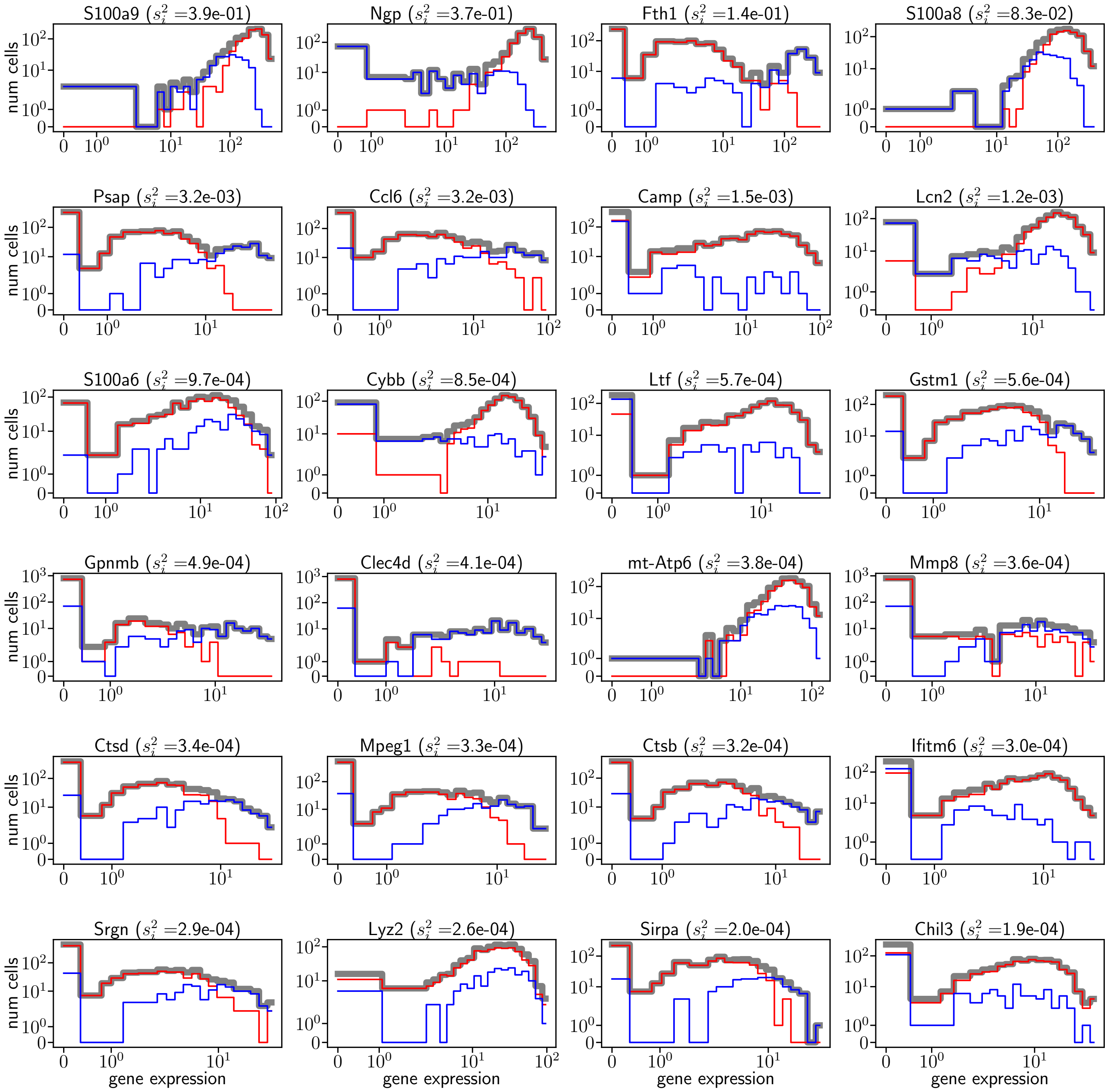

Fig. S13. Distribution of gene expression for genes with the highest absolute weight in $\vec{s}_m$. Red and blue indicate number of cells in each cluster (as in Fig. 5C) while gray indicates total.

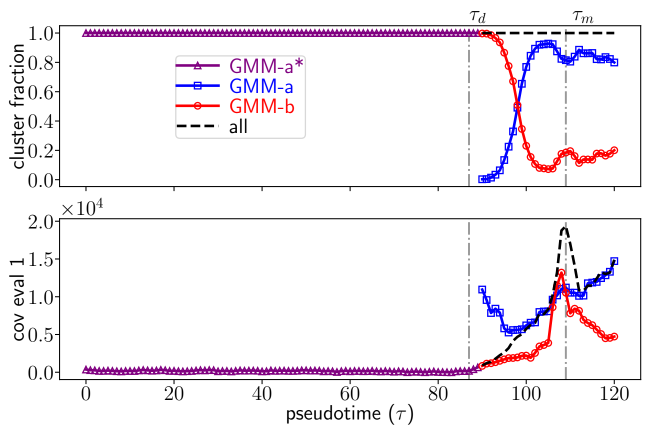

Fig. S14. Top: Fraction of cells in each of the two GMM clusters. Prior to the split between clusters, all cells are classified as GM M -a. Bottom: Covariance eigenvalue at pseudotimes f or t he f ull dataset (black) and each of the GMM clusters (red and blue). Dot -dashed vertical l ines i ndicate τ_d and τ_m.

Development • Supplementary information

Appendix S1

1 The relationship between the Jacobian and the Covariance

Here, we outline the methodological framework that enables characterizing cell-fate transitions directly from scRNA-seq snapshots of a cell's transcriptomic state. A scRNA-seq measurement yields a transcriptomic matrix, where each row is a different cell and each column is a different gene (Fig. S1, left). This data is often visualized via dimensionality reduction algorithms, that reduce the $25,000$ dimensional gene space to two or three axes of variation, and sorted via parametric curve fitting tools, that show how the cells vary as a function of a control parameter, such as developmental time (pseudotime). Thus, one can compute statistics, such as the covariance C of the genes at a given pseudotime window.

Assuming that the underlying biochemical processes (1) are stochastic and Markovian and (2) occur at significantly faster timescales (seconds to minutes) than the timescales over which transitions in cellular fates are observed (hours to days), then the local time evolution of a cell's transcriptomic profile is controlled by a single matrix, the Jacobian ($\mathbf{J}$), where $J_{ij} = \partial \dot{g}_i / \partial g_j$ is the effect of the amount of gene j on the dynamics of gene i (Fig. S1, center). While $\mathbf{J}$, in general, changes with pseudotime, it relates to the covariance of gene expression at that pseudotime $\mathbf{C}$ through the continuous-time Lyapunov equation [4],

$$\mathbf{JC} + \mathbf{CJ}^T + \mathbf{D} = 0 \tag{S1}$$

where $\mathbf{D}$ is the expected noise amplitude for individual genes and their interactions (derivation in Methods: Continuous time Lyapunov equation for transcriptomic matrices) [5]. An important result from this relationship is that in the vincinity of bifurcations, the most salient properties of $\mathbf{J}$, corresponding to its eigen-decomposition, are inferrable from the eigendecomposition of $\mathbf{C}$.

We demonstrate the intuition behind the Eqn. S1 using a one-dimensional toy-model (Fig. S1, right). The slope of the potential function, drawn in red, provides the deterministic features of the system's dynamics. Parameter regimes ($a \ll 0$ and $a \gg 0$) where the potential has highly convex curvature exhibit stable fixed points, while parameter regimes near the bifurcation ($a \sim 0$), that have much flatter curvature, exhibit instability. Stochastic simulations of the system (drawn as open circles in Fig. S1– color corresponds to the value of the control parameter) demonstrate that owing to the reduction in curvature of the underlying potential, the data is spread maximally near the bifurcation, and narrows on either side of it.

This simple one-dimensional toy model captures the essence of the ideas used in this paper. If a complex high-dimensional dynamical system undergoes a bifurcation, then in its vicinity there must be, by definition, some direction in the high-dimensional space with greatly enhanced fluctuations. Thus bifurcations, and regions of multistability, can be located by finding the points along a developmental trajectory in transcriptomic space where the covariance eigenvalue spectrum is dominated by a single principle mode. Moreover, the direction of those fluctuations (the corresponding covariance eigenvector) is equivalient to the soft direction along which the system bifurcates (the corresponding eigenvector of the Jacobian), even in the $25,000$ dimension transcriptomic space.

2 Methodological relationship to Dynamical Network Biomarkers

Chen et al. [6], developed the concept of a dynamical network biomarker (DNB), a group of genes that drive a critical transition and are detectable from high dimensional gene expression datasets. In particular, they define an indicator function

$$I = \frac{SD_d \cdot |PCC_d|}{|PCC_o|} \tag{S2}$$

where SD_d is the average standard deviation of genes in the DNB, PCC_d is the average correlation coefficient between genes in the DNB, and PCC_o is the average correlation coefficient between genes in the DNB and genes outside the DNB [6]. At a critical state transition, or bifurcation, I is predicted to diverge, because SD_d and $|PCC_d|$ become large, while $|PCC_o|$ becomes small. Mathematically, the genes in the DNB correspond to those that have non-zero weight in the direction of the transition, i.e., $\vec{p}_d^{\,i} \neq 0$, where $\vec{p}_d$ is the principal eigenvector of the Jacobian, while genes outside of the DNB have $\vec{p}_d^{\,i} = 0$. This prediction is qualitatively similar, but not the same as Eqn. 2. In particular, while both SD_d and ω_1 increase at a bifurcation, they are not equivalent, as SD_d measures the variance of each individual gene, while ω_1 measures the variance across all genes, and therefore accounts for corrections to the total variance due to covariances between genes in the network. Therefore, for bifurcation detection, we focus solely on ω_1, instead of incorporating correlations into the indicator as in Eqn. S2.

As for determining which gene relationships are critical for the bifurcation, we take a similar approach to Refs. [6, 7], in focusing on the correlations that approach ± 1 at the bifurcation. This is justified via Eqn. 4, which yields that $R_{ij} \to \pm 1$ if $\vec{p}_d^{\,i} \neq 0$ and $\vec{p}_d^{\,j} \neq 0$. Interestingly, while we derived Eqn. 4 via the eigendecomposition of the covariance matrix, Refs. [6, 7] derived the same result form the covariance matrix itself, providing additional support to this method.

Development • Supplementary information

3 Bifurcations possibilities from two mutually inhibiting genes

At steady state, Eqn. 5 satisfies the quintic polynomial

$$g_1 = \frac{m_1/k_D}{\left(\frac{m_2/k_D}{g_1^2+1}\right)^2 + 1} \tag{S3}$$

which, depending on the parameter values, can have one real solution that is an attractor (e.g., if $m_{1,2} = 1$ and $k_D = 1$) or three real solutions, two attractors (nodes) and one repellor (saddle) (e.g., $m_{1,2} = 1, k_D = 1/3$). By examining the null clines,

$$g_1(g_2) = \frac{m_1/k_D}{g_2^2 + 1} \tag{S4}$$

$$g_1(g_2) = \sqrt{\frac{m_2}{k_D g_2} - 1} \tag{S5}$$

it can be deduced that varying m_1, while fixing τ and m_2 can yield a saddle-node bifurcation, as Eqn. S4 moves vertically while Eqn. S5 does not, allowing for either node to merge with the saddle (Fig. S2A).

Conversely, varying k_D, while fixing $m_{1,2}$ and m_2, can yield a pitchfork bifurcation, as both null clines move, such that above the bifurcation value, all three real solutions remain (Fig. S2B). Solving Eqn. S3 computationally via the Python function numpy.roots and plotting the real solutions (Fig. S2C-D) yields the bifurcations used in Fig. 2 and Fig. S5 [8].

4 Resampling principal eigenvalue

Given the transcriptomic matrix $\mathbf{G} = \left\{\vec{g_1}^T, \vec{g_2}^T, \ldots \vec{g_{n_g}}^T\right\}$, where $\vec{g_i} = \{G_{1,i}, G_{2,i}, \ldots, G_{n_c,i}\}$ and $G_{i,j}$ is the expression of the jth gene in the ith cell, we generate a null sample $\mathbf{G}^{\text{null}}$ by drawing each of its entries $G^{\text{null}}_{i,j}$ randomly, with replacement, from $\vec{g_i}$. In Fig. S10Fig. 2,Fig. 3, we compute the principal covariance eigenvalue ω_1^{null} for each of $n_s = 20$ samples, and compare this null distribution against the principal covariance eigenvalue of the data ω_1. This resampling technique has little impact on ω_1 for unimodal distributions as the scale of ω_1 is still determined by the system's noise (Fig. S3 left and right), but significantly decreases ω_1 for multimodal distributions (Fig. S3 center) since the structure of the multimodality is scrambled; thus we found it was an effective method for determining if a spike in ω_1 is due to multimodality or increased noise.

5 Noise induced transitions

To determine if a non-bifurcating noise-induced transition model [9, 10] could yield a similar covariance eigenvalue signature to a bifurcation, we ran the 102 gene network model (Fig. 2A) in a regime of the dynamical system that had two fixed-points ($m_{1,2} = 1$, $k_D = 1/3$) at varying noise scales s (see Fig. S2 and Eqn. 22 for details). To ensure a transition, we initialized all cells to populate the fixed point with higher g_1. We found that for low noise values ($1/s \leq 0.01$) the cells stayed near their initial fixed point, yielding a unimodal distribution for g_1 (Fig. S6A) and low principal covariance eigenvalue (Fig. S6B) while for high noise values ($1/s \geq 0.02$) the cells visited both fixed points, yielding a bimodal distribution for g_1, and a high principal covariance eigenvalue.

6 Effect of small errors

To better understand why the difference between ω_1 and its corresponding null was significantly more apparent at τ_m than τ_d (Fig. 3C), we exmained how small errors in the model parameters effect bifurcations. Specifically, we simulated the GRN model (Eqn. 5) with different amounts of error in other parameters. For the saddle-node bifurcation, in which m_1 is varied while τ_D and m_2 remain fixed, we perturbed m_2 by small amounts from its bifurcation value $m_{2_c} = 3$. We found (Fig. S8A) that the bifurcation was still largely detectable, and its eigenvalue still well distinguished from its null (Fig. S8B), at these small errors. For the pitchfork bifurcation, in which k_D is varied while m_1 and m_2 remain fixed, we perturbed m_1 by small amounts from its bifurcation value of $m_{1_c} = 1$. In this case, we found that the small perturbations biased the bifurcation toward one of the branches (Fig. S8C). This bias significantly reduces the difference ω_1 and its corresponding null (Fig. S8D). Our analysis suggests that small errors in the one-to-many bifurcating dynamical systems that appears present at τ_D may prevent it from being easily detectable, even when similar sized errors do not obscure the one-to-one bifurcation at ω_1.

7 Pseudotime inference

7.1 Algorithm for generating the pseudotime labels in Weinreb et al

SPRING (x-y) positions, cell type annotations, and pseudotime ranks for the data presented in Fig. 3A-B were downloaded from `https://github.com/AllonKleinLab/paper-data/tree/master/Lineage_tracing_on_transcriptional_landscapes_`

Development • Supplementary information

`links_state_to_fate_during_differentiation`. The algorithms to generate these values are described in detail in Ref. [3] (Supplementary Materials) and recapitulated here for completeness. Given the full *in-vitro* hematopoiesis transcriptomic matrix (all cells and all genes), the SPRING positions in Fig. 3A plot were generated using the following procedure.

1. A filtered transcriptomic matrix was generated which did not include genes that
   (a) had low variability as determined via the filter_genes function with parameters (85,3,3) from `https://github.com/AllonKleinLab/SPRING_dev/blob/master/data_prep/spring_helper.py` [1].
   (b) correlated highly ($R > 0.1$) across all cells with any of the following cell cycle genes: Ube2c, Hmgb2, Hmgn2, Tuba1b,Ccnb1, Tubb5, Top2a, and Tubb4b.
2. The top 50 principal components (PC) of the filtered transcriptomic matrix were computed.
3. 40,000 of the cells were selected randomly, and a k-nearest-neighbors (KNN) graph between those cells was constructed using the top 50 PC of the filtered transcriptomic matrix and k=4.
4. X-Y positions of these 40,000 cells were generated using the ForceAtlas2 algorithm with 500 steps [11].
5. Positions for each of the remaining 90,887 cells were computed as the average position of their 40 nearest neighbors (in the 50-PC space) among the initial 40,000 cells.

Cells were annotated with their cell types (cluster annotation in Fig. 3A) based on their position in the SPRING plot and their expression (terminal cell fates) or lack of expression (pluripotent) of pre-selected marker genes. Specifically the marker genes used to determine if cells were neutrophils were S100a9, Itgb2l, Elane, Fcnb, Mpo, Prtn3, S100a6, S100a8, Lcn2, and Lrg1.

Neutrophil pseudotime rank was then determined by smoothly interpolating between cells in the pluripotent and neutrophil clusters. The interpolation method used throughout this procedure is an iterative, diffusive process defined as

$$\begin{aligned} S_0(\mathbf{X}, b, i, k) =& \vec{x}_i \\ S_n(\mathbf{X}, b, i, k) =& b S_{n-1}(\mathbf{X}, b, i, k) \\ &+ \frac{1-b}{k} \sum_{j \in K_k(i)} S_{n-1}(\mathbf{X}, b, j, k) \end{aligned} \tag{S6}$$

where $\vec{x}_i$ is a vector quantity defined for cell i, $\mathbf{X} = \{\vec{x}_1, \vec{x}_2, \ldots, \vec{x}_{n_c}\}$ is the matrix of this quantity for all cells, $K_k(i)$ are the cell indices of the k nearest neighbors of cell i, $n > 0$ is the number of iterations, and b is the neighbor weight (low b and high n both yield high diffusion) [12]. The pseudotime ranking procedure is:

1. Cells are identified to be part of the neutrophil trajectory
   (a) Let $\vec{t}_i$ be an indicator vector for the cell type of i; i.e. $t_{ij} = 1$ if cell i is type j and 0 otherwise. Let $\mathbf{T} = \{\vec{t}_1, \vec{t}_2, \ldots, \vec{t}_{n_c}\}$ be the corresponding matrix for all cells.
   (b) Let $\mathbf{K}_{100}$ be the k-nearest-neighbor graph between cells for $k = 100$ using the top 50 PC.
   (c) Let $\hat{t}_i = S_{250}(\mathbf{T}, 0.1, i, 100)$ be the smooth cell type indicator.
   (d) Let $z_i = \sum_j a_j \hat{t}_{ij}$ be the weighted average cell type $\hat{t}_i$ where the weights for each cell type (j) are

$$a_j = \begin{cases} 0.1 & \text{if neutrophil or pluripotent} \\ -2 & \text{if megakaryocyte} \\ -1 & \text{otherwise} \end{cases} \tag{S7}$$

   (e) Let $\vec{c}_i$ be a neutrophil trajectory indicator such that $\vec{c}_i = \{1\}$ if $z_i > Q_{0.6}(z)$ and $\{0\}$ otherwise, where $Q_{0.6}(z)$ is the 60th quantile of z. Let $\mathbf{C} = \{\vec{c}_1, \vec{c}_2, \ldots, \vec{c}_{n_c}\}$.
   (f) Let $\hat{c}_i = S_{50}(\mathbf{C}, 0.1, i, 100)$ be the smoothed neutrophil trajectory indicator.
   (g) Cells were considered part of the neutrophil trajectory if $\hat{c}_i > Q_{0.6}(\hat{c})$ where $Q_{0.6}(\hat{c})$ is the 60th percentile of $\hat{c}$.
2. The $61,310$ cells identified as part of the neutrophil trajectory are sorted
   (a) Let $\vec{p}_i = \{1\}$ if a cell in the trajectory is pluripotent and 0 otherwise; i.e., it is an indicator for pluripotency. $\mathbf{P} = \{\vec{p}_1, \vec{p}_2, \ldots, \vec{p}_{n_c}\}$ is the corresponding matrix for all cells in the trajectory.
   (b) Let $\hat{p}_i = S_{300}(\mathbf{P}, 0.1, i, 100)$ be the smoothed pluripotency indicator.
   (c) The pseudotime of cell i is the rank (largest to smallest) of $\hat{p}_i$ among all $\hat{p}$.

7.2 Pseudotime inference in the absence of metadata

To test if the neutrophil bifurcation characterization was dependent on the choice of pseudotime algorithm, we used the Slingshot algorithm [2] to compute the pseudotime of each cell for its trajectory from the undifferentiated cluster to each of the terminal fate clusters. The input to Slingshot were the cells' cluster labels and their SPRING coordinates, and the output was a probability, or weight, that a cell belonged to each undifferentiated-to-terminal-fate trajectory, as well as its pseudotime along that trajectory. In Fig. S11A, we show the pseudotime of all cells that had $weight > 0$ for belonging to the trajectory that led from undifferentiated cells toward neutrophils. Unlike the pseudotime method described in Section 7.1, the origin of the trajectory does not coincide with the earliest sequenced cells, as time of sequencing and clonal barcode data could not be input to Slingshot. Nevertheless, we obtain a clear bifurcation signature in the principal covariance eigenvalue (Fig. S11B) at the point where promyelocyte gene expression decreases to 0 and myelocyte marker gene expression become maximal (Fig. S11C). This result supports our belief that the bifurcation characterization does not depend on the specific pseudotime calculation.

8 Determining the eigenvectors for analysis

In order to analyze the neutrophil trajectory in a native-space, we chose eigenvectors that were characteristic of the dynamics. Since τ_m coincides with a well defined eigenvalue peak in the neutrophil trajectory, it was natural to use $\vec{s}(\tau_m)$ to aid in visualizing the trajectory and further probe mechanisms. However, $\tau = 0$ and τ_d coincide with transition points between states (Fig. 5B), and mark the beginning of specific dynamics (i.e., the eigenvalue remaining constant, or increasing), and it the lower correlation on the edges of the blocks in Fig. 5B suggests that the eigenvectors at those points had not equiilibrated to their new positions. Therefore, we define $\tilde{\tau}_0$ and $\tilde{\tau}_d$ as the pseudotime bins with the eigenvector closest to the eigenvector at all other pseudotimes in that range, i.e.,

$$\tilde{\tau}_0 = \underset{0<=\tau<\tau_d}{\arg\min} \sum_{t=0}^{\tau_d-1} ||\vec{s}(\tau) - \vec{s}(t)||^2 \tag{S8}$$

$$\tilde{\tau}_d = \underset{\tau_d<=\tau<\tau_m}{\arg\min} \sum_{t=\tau_d}^{\tau_m-1} ||\vec{s}(\tau) - \vec{s}(t)||^2 \tag{S9}$$

and use these pseudotimes for downstream analysis.

9 Identifying clusters via Gaussian Mixture Models

As the distribution of gene expression projected onto $\vec{s}(\tau_m)$ exhibited bimodality (Fig. 5E, Fig. S12B), we used a Gaussian Mixture Model to separate the two modes. Specifically, we fit $\mathbf{G}(\tau_m)$, the normalized gene expression matrix at τ_m to a two component Gaussian Mixture Model using the `mixture.GaussianMixture` function from the `Python` package `scikit-learn` with $n\_components = 2$ and all other parameters set to their default [13]. We then used the `predict` function of our trained model to generate cluster labels for cells at all pseudotimes. We found that cells were predicted to belong to the same cluster (GMM-a) for $\tau \lesssim \tau_d$ (purple in Fig. 5F and Fig. S14). For $\tau \gtrsim \tau_d$, cells were split between the two clusters (red and blue in Fig. 5F and Fig. S14).

References

[1] Caleb Weinreb, Samuel Wolock, and Allon M Klein. Spring: a kinetic interface for visualizing high dimensional single-cell expression data. *Bioinformatics*, 34(7):1246–1248, 2018.

[2] Kelly Street, Davide Risso, Russell B Fletcher, Diya Das, John Ngai, Nir Yosef, Elizabeth Purdom, and Sandrine Dudoit. Slingshot: cell lineage and pseudotime inference for single-cell transcriptomics. *BMC genomics*, 19(1):477, 2018.

[3] Caleb Weinreb, Alejo Rodriguez-Fraticelli, Fernando D Camargo, and Allon M Klein. Lineage tracing on transcriptional landscapes links state to fate during differentiation. *Science*, 367(6479), 2020.

[4] Nicolaas Godfried Van Kampen. *Stochastic processes in physics and chemistry*, volume 1. Elsevier, 1992.

[5] Makito Oku and Kazuyuki Aihara. On the covariance matrix of the stationary distribution of a noisy dynamical system. *Nonlinear Theory and Its Applications, IEICE*, 9(2):166–184, 2018.

[6] Luonan Chen, Rui Liu, Zhi-Ping Liu, Meiyi Li, and Kazuyuki Aihara. Detecting early-warning signals for sudden deterioration of complex diseases by dynamical network biomarkers. *Scientific reports*, 2(1):1–8, 2012.

[7] Mitra Mojtahedi, Alexander Skupin, Joseph Zhou, Ivan G Castaño, Rebecca YY Leong-Quong, Hannah Chang, Kalliopi Trachana, Alessandro Giuliani, and Sui Huang. Cell fate decision as high-dimensional critical state transition. *PLoS biology*, 14(12):e2000640, 2016.

[8] Charles R. Harris, K. Jarrod Millman, St'efan J. van der Walt, Ralf Gommers, Pauli Virtanen, David Cournapeau, Eric Wieser, Julian Taylor, Sebastian Berg, Nathaniel J. Smith, Robert Kern, Matti Picus, Stephan Hoyer, Marten H. van Kerkwijk, Matthew Brett, Allan Haldane, Jaime Fern'andez del R'ıo, Mark Wiebe, Pearu Peterson, Pierre G'erard-Marchant, Kevin Sheppard, Tyler Reddy, Warren Weckesser, Hameer Abbasi, Christoph Gohlke, and Travis E. Oliphant. Array programming with NumPy. *Nature*, 585(7825):357–362, September 2020.

[9] Caleb Weinreb, Samuel Wolock, Betsabeh K Tusi, Merav Socolovsky, and Allon M Klein. Fundamental limits on dynamic inference from single-cell snapshots. *Proceedings of the National Academy of Sciences*, 115(10):E2467–E2476, 2018.

[10] Peijie Zhou, Shuxiong Wang, Tiejun Li, and Qing Nie. Dissecting transition cells from single-cell transcriptome data through multiscale stochastic dynamics. *bioRxiv*, 2021.

[11] Mathieu Jacomy, Tommaso Venturini, Sebastien Heymann, and Mathieu Bastian. Forceatlas2, a continuous graph layout algorithm for handy network visualization designed for the gephi software. *PloS one*, 9(6):e98679, 2014.

[12] Betsabeh Khoramian Tusi, Samuel L Wolock, Caleb Weinreb, Yung Hwang, Daniel Hidalgo, Rapolas Zilionis, Ari Waisman, Jun R Huh, Allon M Klein, and Merav Socolovsky. Population snapshots predict early haematopoietic and erythroid hierarchies. *Nature*, 555(7694):54–60, 2018.

[13] F. Pedregosa, G. Varoquaux, A. Gramfort, V. Michel, B. Thirion, O. Grisel, M. Blondel, P. Prettenhofer, R. Weiss, V. Dubourg, J. Vanderplas, A. Passos, D. Cournapeau, M. Brucher, M. Perrot, and E. Duchesnay. Scikit-learn: Machine learning in Python. *Journal of Machine Learning Research*, 12:2825–2830, 2011.

Development • Supplementary information
