# Supplementary material for: A dynamical systems treatment of transcriptomic trajectories in hematopoiesis
Source: Development. 2023 Jun 1;150(11):dev201280. doi: 10.1242/dev.201280 (PMC10281553; doi:10.1242/dev.201280)
Supplement: Supplementary file 1 [file develop-150-201280_review_history.pdf]

A dynamical systems treatment of transcriptomic trajectories in hematopoiesis

Simon L. Freedman, Bingxian Xu, Sidhartha Goyal and Madhav Mani
DOI: 10.1242/dev.201280

Editor: François Guillemot

Review timeline
Original submission: 9 September 2022
Editorial decision: 24 October 2022
First revision received: 23 March 2023
Accepted: 11 April 2023

Original submission

First decision letter

MS ID#: DEVELOP/2022/201280

MS TITLE: A dynamical systems treatment of transcriptomic trajectories in hematopoiesis

AUTHORS: Simon Freedman, Bingxian Xu, Sidharth Goyal, and Madhav Mani

I have now received all the referees' reports on the above manuscript, and have reached a decision. The referees' comments are appended below, or you can access them online: please go to BenchPress and click on the 'Manuscripts with Decisions' queue in the Author Area.

As you will see, the referees express considerable interest in your work, but one reviewer has some significant criticisms and recommends a substantial revision of your manuscript before we can consider publication. If you are able to revise the manuscript along the lines suggested, I will be happy receive a revised version of the manuscript. Your revised paper will be re-reviewed by one or more of the original referees, and acceptance of your manuscript will depend on your addressing satisfactorily the reviewers' major concerns. Please also note that Development will normally permit only one round of major revision. If it would be helpful, you are welcome to contact us to discuss your revision in greater detail. Please send us a point-by-point response indicating your plans for addressing the referee's comments, and we will look over this and provide further guidance.

Please attend to all of the reviewers' comments and ensure that you clearly highlight all changes made in the revised manuscript. Please avoid using 'Tracked changes' in Word files as these are lost in PDF conversion. I should be grateful if you would also provide a point-by-point response detailing how you have dealt with the points raised by the reviewers in the 'Response to Reviewers' box. If you do not agree with any of their criticisms or suggestions please explain clearly why this is so.

Reviewer 1

Advance summary and potential significance to field

In this manuscript, the authors seek to identify signatures of cell differentiation dynamics from single-cell RNA sequencing data. Specifically, drawing on synthetic data from a stochastic

© 2023. Published by The Company of Biologists under the terms of the Creative Commons Attribution License (https://creativecommons.org/licenses/by/4.0/).

dynamical model and published experimental data on hematopoietic cell lineages, they argue that the local correlation structure of transcriptomic data can reveal bifurcations in the underlying dynamics.

Extracting dynamical models from single-cell data, to move from descriptive analyses of cell trajectories to a more predictive understanding of how they are shaped, and to ask how aptly they are captured by landscape metaphors, is of great interest. Based on the current presentation of this study, however, I remain unclear about the extent to which it differs from previous work, and to which it illuminates the experimental system considered.

Comments for the author

1. The point that the correlation structure of single-cell data can reveal bifurcations has been made previously in ref. 22 (as I understand it, in rather similar terms, as a generic property of a dynamical system, see ref. 22, S2 Appendix, section B3). The authors should better discuss how their treatment differs from that previous work.

2. I am a bit puzzled by the premise that the cell state distributions are near steady state as a control parameter is slowly changed (l. 47-59). I agree that some of the underlying molecular processes occur on faster time scales, but I find the assumption of a near-steady-state hard to reconcile with the intuitive picture of bifurcating trajectories in a Waddington landscape. Is the time scale of cell fate changes - hours or days (cf. l. 52-53) - not the same thing as the time scale of cell state changes? Does this assumption of near-steady-state not disallow memory in the cell trajectories, and therefore the early fate biases that recent work (e.g. Wang et al, CoSpar identifies early cell fate biases from single-cell transcriptomic and lineage information, 2022) has sought to detect in single-cell data? Although this assumption may provide a simple way to argue that correlations provide a signature of bifurcations, I believe it needs further discussion.

3. Relatedly, one could interpret Waddington's landscapes to imply that the pseudo-time extracted from the data is a dimension of intrinsic cell dynamics (the downward slope of valleys running into saddles), rather than an external control parameter. In the authors' view, what is the biological correlate of this slowly-varying control parameter? Along the same line, one can wonder if it is fair to compare the statistics of cells sorted by control parameter (as for the synthetic data, cf. Fig 2) with cells sorted by pseudo-time (as for the experimental data, cf. Fig 3). How would the results change if the authors extracted a pseudo-time from the synthetic data and computed the correlation structure vs. pseudo-time?

4. I am not fully convinced by some of the connections that authors seek to establish between their statistical analysis of single-cell data and the biological events of cell differentiation in hematopoietic lineages. The authors claim that the sign of the difference between the actual correlations and a null model (Fig 3C) are indicative of a regime crossover (l. 429), but how are we to tell that these small differences are significant? Can their model illuminate how such differences could arise? Based on Fig 2 Supp 4, the correlations increase faster than those from shuffled data at the approach to a pitchfork bifurcation. Then, how is the trend in experimental data indicative of a one-to-many state transition (l. 435-436)? And, given that the analysis is restricted to the neutrophil lineage, how would such a one-to-many state transition fit into the lineage tree depicted in Fig 4A? Lastly, the time courses of fate marker genes in Fig 4C suggest that a state transition from promyelocyte to myelocyte (the one state transition that is expected along the neutrophil branch according to Fig 4A) may be under way as early at tau_d, but the signature of a bifurcation (based on the difference between correlations and their shuffled counterparts) is only evident around tau_m. How can this be reconciled with a model in which the dynamics of a subset of genes driving a state transition governs the correlation structure of transcriptional states?

5. Maybe most informative is the analysis of the correlation eigenvectors over time, which uncovers a finer structure to neutrophil differentiation trajectories, as displayed in Fig 6. In particular, the dominant eigenvector appears to switch rather abruptly at the times tau_d and tau_m. To complement this representation of the dominant eigenvectors vs. time, it could be useful to show the variances along different directions vs. time (e.g. alongside the projected distributions in Fig 6 Supp 1), to see how strongly one direction dominates over others at different times. I do have one question about the choice of the three eigenvectors for this analysis: given that the dominant

© 2023. Published by The Company of Biologists under the terms of the Creative Commons Attribution License (https://creativecommons.org/licenses/by/4.0/).

eigenvector appears relatively stable in the interval between tau_d and tau_m, would it not be natural to choose eigenvectors in this and other intervals where it is stable (within blocks), rather than the eigenvectors at the transition points tau_d and tau_m (at block boundaries)? Would this not yield eigenvectors that are more weakly correlated (vs. .67 for the vectors at tau-d and tau_m, cf. l. 620), and therefore better separate the cell states over time? Currently, the representation in Fig 6C appears to be dominated by 'motion' along a diagonal in the plane spanned by the two chosen vectors. If different regions (the colored clusters in Fig 6F) correspond to different stages along the neutrophil differentiation path (l. 687), does this is mean that a different assignment of the pseudo-time could collapse the transcriptional states onto a 1D trajectory, or is the dynamics truly multidimensional (maybe this is a question about the extent to which the eigenvectors align with a vector tangent to the mean trajectory of cells vs. pseudo-time)?

Taken together, my evaluation is that this manuscript touches on important and interesting questions, but, in its present state, does not sufficiently demonstrate the novelty of the proposed approach and its ability to deliver new insights into a concrete instance of cell differentiation dynamics. If the connection between correlations and bifurcations is not the most novel aspect of this work, as is my impression, I would encourage the authors to acknowledge this more clearly, to reconsider the claims made on the basis of eigenvalues alone (point 4 above), and put more weight on the analysis of the eigenvectors vs. time, maybe reexamining the choice of the eigenvectors chosen to project the trajectories (point 5).

Reviewer 2

Advance summary and potential significance to field

The manuscript by Freedman et al. uses fundamental properties of dynamical systems at a bifurcation point to assess whether differentiation events in RNAseq data occur due to passing through a bifurcation. Specifically, the authors use the fact that signatures of a bifurcation in the Jacobian of a dynamical model correspond to analogous signatures in the covariance matrix. Because the covariance matrix is accessible from the data, this allows bifurcations to be detected without assuming a dynamical model. The authors validate the approach on data generated by a model with known saddle and pitchfork bifurcations, and then apply the approach to published RNAseq data on hematopoietic differentiation. The application identifies a one-to-many (pitchfork-like) transition near markers of a putative one-to-many differentiation event in developmental time, and a one-to-one (saddle-like) transition near markers of a putative one-to-one differentiation event in developmental time. Properties of the covariance matrix near the saddle point reveal known and new gene-gene connections, and the structure of the principal eigenvector reveals a principled and interpretable dimensional reduction of the developmental dynamics.

The study is rich with results but elegant and clear. Despite the fact that the problem is complex, the focus on a few fundamental mathematical results, and the validation with a clear toy example, make the work suitable for a broad, biologically-minded audience. The fact that the approach is generic, interpretable, and more efficient and requires less choice than existing methods, makes it more likely to be broadly adopted. Given the recent explosion in RNAseq data, this gives the work potential for high impact. Overall, I recommend publication after the following minor comments are addressed.

Comments for the author

1. The most glaring question hanging over the results is that in the toy model the increase in the covariance matrix's principle eigenvalue omega_1 at the pitchfork bifurcation is distinguishable from its null model (Fig 2 Supp 4B), whereas with the hematopoiesis data it is not (Fig 3C, black and gray at tau_d). The authors point out this discrepancy themselves, and many other features of their results support the interpretation of tau_d as a one-to-many transition, but an understanding of the discrepancy is still lacking. This leads to two questions:

1a. The authors point out that the difference between the actual and null values of omega_1 changes sign somewhat later than tau_d, and they call this a "regime change." This term is kind of

© 2023. Published by The Company of Biologists under the terms of the Creative Commons Attribution License (https://creativecommons.org/licenses/by/4.0/).

overkill: the sign switch could just as easily be due to the null data decreasing in advance of the peak at tau_m. Moreover, the inset, which is designed to highlight this change, does not add new information, and is in fact misleading: showing a log scale both above and below zero allows one to separate positive and negative values arbitrarily. The authors appear to do just that, making the sign change look like a huge jump. Please remove the inset and tone down the language about the sign change.

1b. It occurred to me that with a pitchfork bifurcation, if the bifurcation trajectory is slightly "off", you do not really get a pitchfork (like an Ising model transitioning through its critical temperature but with a small magnetic field)--you get a "disconnected" pitchfork that is biased to one of the two states. But with a saddle bifurcation, if you are slightly off, you still get a saddle (like an Ising model transitioning through zero magnetic field at two of many subcritical temperatures). This suggests a test in the toy model: if you vary 1/k_D to create the pitchfork, but change one or more the other parameters away from their "ideal" values, does it become harder to distinguish the increase in omega_1 from that in the null model? This would provide mechanistic insight for why a one-to-many transition may be harder to detect via cross-validation than a one-to-one transition.

2. The alternative to bifurcation-based state transitions, namely noise-induced state transitions, was not clear to me when it was first introduced (lines 34-38). It only became somewhat clear later when tested with the toy model. I would suggest either adding a cartoon to Fig 1 or adding a sentence or two at those lines to more clearly paint the picture of noise-induced transitions as a plausible mechanism for differentiation.

First revision

<u>Author response to reviewers' comments</u>

Reviewer 1 Advance Summary and Potential Significance to Field:
In this manuscript, the authors seek to identify signatures of cell differentiation dynamics from single-cell RNA sequencing data. Specifically, drawing on synthetic data from a stochastic dynamical model and published experimental data on hematopoietic cell lineages, they argue that the local correlation structure of transcriptomic data can reveal bifurcations in the underlying dynamics.

Extracting dynamical models from single-cell data, to move from descriptive analyses of cell trajectories to a more predictive understanding of how they are shaped, and to ask how aptly they are captured by landscape metaphors, is of great interest. Based on the current presentation of this study, however, I remain unclear about the extent to which it differs from previous work, and to which it illuminates the experimental system considered.

Reviewer 1 Comments for the Author:
1. The point that the correlation structure of single-cell data can reveal bifurcations has been made previously in ref. 22 (as I understand it, in rather similar terms, as a generic property of a dynamical system, see ref. 22, S2 Appendix, section B3). The authors should better discuss how their treatment differs from that previous work.

We thank the reviewer for highlighting these earlier studies. We have now clarified that these lines of work similarly analyze correlation structure (last paragraph of the Introduction, lines 113-116), and have been previously applied to single-cell data (Results, second to last paragraph, line 203). In Figs 2D and 3C, we compare empirically with this analysis, and find that the Dynamic Network Biomarker (DNB) indicator follows a similar trend to the principal covariance eigenvalue, providing an empirical benchmark for our results. In Supp. Secn S2, we compare analytically with these results and find that they are grounded in a similar theoretical stance.

© 2023. Published by The Company of Biologists under the terms of the Creative Commons Attribution License (https://creativecommons.org/licenses/by/4.0/).

Importantly, we have also expounded upon the differences between the DNB framework, and our own (Results, first section, second to last paragraph, lines 202-210). First, in our framework, it is not necessary to delineate which genes drive the bifurcation, whereas in Ref. 22 and similar works, it is necessary to delineate them (i.e., the DNB). To make this point explicit, we show in Fig. S8B, that while choosing neutrophil marker genes as a DNB yields a similar indicator trend to the principal covariance eigenvalue, randomly sampled genes do not. Second, we focus on the eigen-decomposition of the covariance as a bifurcation indicator, as opposed to correlation coefficients. This is not only computationally simpler, but also enables our exploration of the covariance eigenvectors, which we found (Results, last section) can elucidate bifurcation mechanisms, and aid in the interpretation of the bifurcation (e.g., by highlighting bimodality). We thank the referee for ensuring that we both acknowledge prior work in the community and precisely identify the advance we present.

2. I am a bit puzzled by the premise that the cell state distributions are near steady state as a control parameter is slowly changed (l. 47-59). I agree that some of the underlying molecular processes occur on faster time scales, but I find the assumption of a near-steady-state hard to reconcile with the intuitive picture of bifurcating trajectories in a Waddington landscape. Is the time scale of cell fate changes - hours or days (cf. l. 52-53) - not the same thing as the time scale of cell state changes? Does this assumption of near-steady-state not disallow memory in the cell trajectories, and therefore the early fate biases that recent work (e.g. Wang et al, CoSpar identifies early cell fate biases from single-cell transcriptomic and lineage information, 2022) has sought to detect in single-cell data? Although this assumption may provide a simple way to argue that correlations provide a signature of bifurcations, I believe it needs further discussion.

We thank the reviewer for raising a set of very deep and relevant issues here, which we have sought to clarify in our updates to our manuscript.

In response to " Is the time scale of cell fate changes - hours or days (cf. l. 52-53) - not the same thing as the time scale of cell state changes? ", our study is fundamentally based on a hypothesis that there exists a qualitative separation of timescales between fast and slow subsets of the dynamics. The dynamics of the slow system are what we postulate give rise to the developmental time that emerges, acting as a control parameter - for example like a morphogen gradient in the context of fly embryonic development. The dynamics of the fast system are then assumed to be "adiabatic", and in equilibrium with the instantaneous value of the slowly varying control parameters - in the context of fly embryos it would be the dynamics of downstream expression that defines the boundaries between cells. We have clarified this hypothesis (3rd paragraph of the introduction, lines 84-97) by stating more explicitly that cell fates can be associated with transcriptomic steady states, which are the result of fast underlying dynamics , but governed by a slow varying parameter, such as morphogen concentrations.

In response to "Does this assumption of near-steady-state not disallow memory in the cell trajectories, and therefore the early fate biases that recent work (e.g. Wang et al, CoSpar identifies early cell fate biases from single-cell transcriptomic and lineage information, 2022) has sought to detect in single-cell data? ", we do not believe that our assumptions are inconsistent with the beautiful results reported by Wang et al.. The reviewer is correct that in case of an ideal pitchfork bifurcation there is no prior state data that could indicate which branch a particular cell (or its descendents) would traverse - as such that decision is stochastic in the model. However, the far more generic state of a pitchfork bifurcation is where the underlying symmetry is already broken, rather than spontaneously broken - this is referred to as an imperfect or broken pitchfork. In this case each cell/lineage has some parameters that precondition it to traverse specific branches of the pitchfork. A simple physical analogy is helpful here - a coffee straw under a uniaxial load can buckle in any direction, however, in a presence of small defects the symmetry is broken and the straw will deterministically buckle in a predictable single direction. A condensed matter analogy is one where a very small external magnetic field will bias the up or down spin at the onset of magnetization.

Mathematically, the normal form to study such generic transitions is $x = h + rx - x^3$. The reviewer will recognize that when h=0 we recover the one-dimensional normal form for a pitchfork bifurcation. When h is nonzero the pitchfork is broken. h is referred to as an imperfection parameter. The figure here corresponds to these two scenarios

© 2023. Published by The Company of Biologists under the terms of the Creative Commons Attribution License (https://creativecommons.org/licenses/by/4.0/).

Figure provided for reviewer has been removed. It showed Fig. 9 from Kitanov et al. (2013) Double zero bifurcation with Huygens symmetry. Dynamics of Continuous, Discrete and Impulsive Systems Series A: Mathematical Analysis. 20(2):197-226.

Fascinatingly, perhaps the early biases that Wang et al. are inferring are precisely these imperfections that pre-condition cells, and their descendants, to traverse distinct branches of a one-to-many transition. We therefore think that the assumption of steady-state can still be preserved and reasonable, while still allowing for early fate biases of the kind reported by Wang et al. For instance, in the context of hematopoiesis, we can imagine stem cells "see" a gradient of some signaling molecule in the marrow which biases them, and provides the biological analog of h. These "biases" or external magnetic fields that make the dynamics more deterministic, we anticipate, might be generic in biological systems and identifying the nature of these "biases" is an exciting line of investigation. While an extensive analysis is outside the scope of the current manuscript, we believe these biases may contribute to the lack of distinction between null and signal during many of the neutrophil transitions. We have therefore incorporated this discussion into our Results section, when explaining the promyelocyte transition (penultimate paragraph of "Covariance analysis pinpoints…" section, lines 562-570) as well as in our Discussion (3rd paragraph, lines 790-794).

3. Relatedly, one could interpret Waddington's landscapes to imply that the pseudo-time extracted from the data is a dimension of intrinsic cell dynamics (the downward slope of valleys running into saddles), rather than an external control parameter. In the authors' view, what is the biological correlate of this slowly-varying control parameter? Along the same line, one can wonder if it is fair to compare the statistics of cells sorted by control parameter (as for the synthetic data, cf. Fig 2) with cells sorted by pseudo-time (as for the experimental data, cf. Fig 3). How would the results change if the authors extracted a pseudo-time from the synthetic data and computed the correlation structure vs. pseudo-time?

This comment by the reviewer cuts to the heart of fundamental features of the problem. We assume that there are underlying genetic variables that are varying slowly in time, controlling the dynamics of the rest of the genome and its expression. While a goal, at present we do not know how to identify these control variables. One of the central leaps in this paper is to conjecture that whatever the complex and molecular nature of the underlying control parameters, they vary slowly in time, and, thus, the pseudotime parameter is an empirical proxy for their variation; this has also been postulated and used similarly for single-cell data in Chen, et al (A branchpoint on differentiation trajectory is the bifurcating event revealed by dynamical network biomarker analysis of single-cell data, 2018). Though we have no empirical evidence for this, we anticipate that the molecular nature of these control parameters must be related to the functional pools of signaling molecules and ligands that are what in-vitro experiments toggle to elicit fate transitions in a dish (e.g., BMP in inducing neural tissue development in Xenopus). Identifying these is one of our central goals but beyond the scope of the current work. We have clarified that (3rd paragraph, introduction, lines 95-99) that we do not believe pseudotime itself is a control parameter, but rather that it may be used as a readout of one since it follows intrinsic cellular dynamics, which are linked to biological control parameters such as morphogen gradients in a spatial context.

The reviewer's suggestion that we ought to verify whether this approach is legitimate even in the scenario of our toy model is a very good one. We have verified that the results for our synthetic toy model are invariant to whether we use the parameters that elicit the transition or pseudotime itself. We have included this result in our revised manuscript (5th paragraph of Results section "Covariance analysis recovers…", lines 308-332, and Fig S4).

4. I am not fully convinced by some of the connections that authors seek to establish between their statistical analysis of single-cell data and the biological events of cell differentiation in hematopoietic lineages. The authors claim that the sign of the difference between the actual correlations and a null model (Fig 3C) are indicative of a regime crossover (l. 429), but how are we to tell that these small differences are significant? Can their model illuminate how such differences could arise? Based on Fig 2 Supp 4, the correlations increase faster than those from shuffled data at the approach to a pitchfork bifurcation. Then, how is the trend in experimental data indicative of a one-to-many state transition (l. 435-436)? And, given that the analysis is restricted to the

© 2023. Published by The Company of Biologists under the terms of the Creative Commons Attribution License (https://creativecommons.org/licenses/by/4.0/).

neutrophil lineage, how would such a one-to-many state transition fit into the lineage tree depicted in Fig 4A?

We would like to thank the reviewer for asking questions that have made us reflect on our understanding and views on both more fundamental and data-centric aspects of our study. The reviewer is correct that the eigenvalue analysis does not pinpoint a pitchfork style bifurcation in the data. Were there such a one-to-many bifurcation we would expect to see a clearer separation between data and null. However, as the reviewer points out, clearly something about the dynamical system is altered at ? . Indeed, our own concerns about the separation between data and null is what spurred our analysis of the dominant eigenvectors as a function of time. The eigenvector analysis does far more clearly identify a transition of sorts at T_d . However, at present time we are unable to precisely identify the nature of this transition. Referring to a previous response of ours, it might well be the case that what we are seeing are signatures of an imperfect pitchfork bifurcation, which is though a bifurcation of a fundamentally one-to-many nature results in what looks like a one-to-one transition.

To address these considerations, we have made several modifications to our description of the eigenvalue signal. We have removed the phrase "regime crossover" as well as the inset to Fig. 4A, as the scale may have been misleading (Results, lines 516-518) and noted the closeness between the signal and null (lines 521-523). As the reviewer suggested, we used our model to investigate the sensitivity of the difference between the eigenvalue and its corresponding null to small perturbations. We found that in our toy model, the saddle-node bifurcation is significantly less sensitive than a pitchfork bifurcation, and have included this result (Supplemental Section S3.3 "Effect of Small Errors", and Fig. S8). We explain in the manuscript (Results, lines 548-553) this may be the reason that the one to one transition we observed was significantly more distinct from its null than the one to many transition.

Lastly, the time courses of fate marker genes in Fig 4C suggest that a state transition from promyelocyte to myelocyte (the one state transition that is expected along the neutrophil branch according to Fig 4A) may be under way as early at tau_d, but the signature of a bifurcation (based on the difference between correlations and their shuffled counterparts) is only evident around tau_m. How can this be reconciled with a model in which the dynamics of a subset of genes driving a state transition governs the correlation structure of transcriptional states?

Taking on board the reviewers several comments we have more carefully and cautiously speculated as to the nature of the transitions we are seeing signatures of. Taking into consideration the eigenvalue and eigenvector analysis it is clear that there are transitions both at T_d and T_m . We do not however have a single coherent mathematical description for these transitions. The fact that two distinct sets of eigenvectors are identified at T_d and T_m does, however, suggest that the underlying dominant genetic factors aren't fully overlapping. While the reviewer has not suggested in as many words, there is a sense in which the evidence hints that the two transitions are part of a single dynamical event that we still do not have a complete understanding of. However, we would put forward that the approach we have pursued does provide some important useful insight and interpretation. We have therefore incorporated our approach, as well as the possibility that the two transitions are linked, perhaps via fate biases, as a possible explanation for the eigenvalue rise, and its indistinguishability between null and signal during the GMP - promyelocyte transition (Results penultimate paragraph of "Covariance analysis pinpoints…", lines 565-570). We have also noted (lines 562-565) that the marker genes are not necessarily the drivers of the bifurcation, and that their dynamics may be influenced by other factors, thus yielding non-discontinuous dynamics. We are very grateful for the thoroughness and depth of the reviews.

5. Maybe most informative is the analysis of the correlation eigenvectors over time, which uncovers a finer structure to neutrophil differentiation trajectories, as displayed in Fig 6. In particular, the dominant eigenvector appears to switch rather abruptly at the times tau_d and tau_m. To complement this representation of the dominant eigenvectors vs. time, it could be useful to show the variances along different directions vs. time (e.g. alongside the projected distributions in Fig 6 Supp 1), to see how strongly one direction dominates over others at different times. I do have one question about the choice of the three eigenvectors for this analysis: given that the dominant eigenvector appears relatively stable in the interval between tau_d and tau_m, would it not be natural to choose eigenvectors in this and other intervals where it is stable (within blocks), rather

© 2023. Published by The Company of Biologists under the terms of the Creative Commons Attribution License (https://creativecommons.org/licenses/by/4.0/).

than the eigenvectors at the transition points tau_d and tau_m (at block boundaries)? Would this not yield eigenvectors that are more weakly correlated (vs. .67 for the vectors at tau- d and tau_m, cf. l. 620), and therefore better separate the cell states over time? Currently, the representation in Fig 6C appears to be dominated by 'motion' along a diagonal in the plane spanned by the two chosen vectors. If different regions (the colored clusters in Fig 6F) correspond to different stages along the neutrophil differentiation path (l. 687), does this is mean that a different assignment of the pseudo-time could collapse the transcriptional states onto a 1D trajectory, or is the dynamics truly multidimensional (maybe this is a question about the extent to which the eigenvectors align with a vector tangent to the mean trajectory of cells vs. pseudo-time)?

We thank the reviewer for these suggestions. We have performed the suggested analyses (variances along directions in Fig S12 A, and projection onto the tangent vector in Fig S12 C) and found that while these plots did highlight some similarities between the eigenvectors (e.g., the fraction of variance along $s1(T_d)$ is high at T_m), they also showed distinct features (the fraction of variance of $S_1(T_m)$ is only high at (T_m). We have incorporated this analysis and interpretation into our manuscript (Results, third paragraph of "Covariance eigenvectors…", lines 626-640). As the reviewer suggested they were extremely helpful in deciphering the extent to which the two bifurcation eigenvectors differ, and the dimensionality of the bifurcation.

We note that in our submitted manuscript, we had already taken care to use eigenvectors that were not exactly at T_0 and T_D, since as the reviewer pointed out, these might be unstable, but rather at $T_{0,}$ T_D , which were in the middle of their corresponding blocks, as detailed in the Supplemental Section Determining the Eigenvectors for Analysis. We chose to use $s(T_m)$ directly, because it seems more motivated by the Lyapunov formalism (Eq. 3). As these details were unclear, we have incorporated them into the manuscript text (lines 618-623).

Taken together, my evaluation is that this manuscript touches on important and interesting questions, but, in its present state, does not sufficiently demonstrate the novelty of the proposed approach and its ability to deliver new insights into a concrete instance of cell differentiation dynamics. If the connection between correlations and bifurcations is not the most novel aspect of this work, as is my impression, I would encourage the authors to acknowledge this more clearly, to reconsider the claims made on the basis of eigenvalues alone (point 4 above), and put more weight on the analysis of the eigenvectors vs. time, maybe reexamining the choice of the eigenvectors chosen to project the trajectories (point 5).

Taken as a whole we are grateful for the incredibly constructive and deep review. The reviews have not just improved the manuscript but increased the precision with which we now think about the strength and limitations of the abstraction and method we present in the manuscript. In addition, we thank the referee for helping us place our advance in the methodological and biological context of the various studies and approaches presently in the community. In particular, we agree that the significant novelty of the approach is the analyses of the dominant eigenvectors over time and we have altered the emphasis of the manuscript to reflect this.

Reviewer 2 Advance Summary and Potential Significance to Field:
The manuscript by Freedman et al. uses fundamental properties of dynamical systems at a bifurcation point to assess whether differentiation events in RNAseq data occur due to passing through a bifurcation. Specifically, the authors use the fact that signatures of a bifurcation in the Jacobian of a dynamical model correspond to analogous signatures in the covariance matrix. Because the covariance matrix is accessible from the data, this allows bifurcations to be detected without assuming a dynamical model. The authors validate the approach on data generated by a model with known saddle and pitchfork bifurcations, and then apply the approach to published RNAseq data on hematopoietic differentiation. The application identifies a one-to-many (pitchfork-like) transition near markers of a putative one-to-many differentiation event in developmental time, and a one-to-one (saddle-like) transition near markers of a putative one-to-one differentiation event in developmental time. Properties of the covariance matrix near the saddle point reveal known and new gene-gene connections, and the structure of the principal eigenvector reveals a principled and interpretable dimensional reduction of the developmental dynamics.

The study is rich with results but elegant and clear. Despite the fact that the problem is complex, the focus on a few fundamental mathematical results, and the validation with a clear toy example,

© 2023. Published by The Company of Biologists under the terms of the Creative Commons Attribution License (https://creativecommons.org/licenses/by/4.0/).

make the work suitable for a broad, biologically-minded audience. The fact that the approach is generic, interpretable, and more efficient and requires less choice than existing methods, makes it more likely to be broadly adopted. Given the recent explosion in RNAseq data, this gives the work potential for high impact. Overall, I recommend publication after the following minor comments are addressed.

Reviewer 2 Comments for the Author:
1. The most glaring question hanging over the results is that in the toy model the increase in the covariance matrix's principle eigenvalue omega_1 at the pitchfork bifurcation is distinguishable from its null model (Fig 2 Supp 4B), whereas with the hematopoiesis data it is not (Fig 3C, black and gray at tau_d). The authors point out this discrepancy themselves, and many other features of their results support the interpretation of tau_d as a one-to-many transition, but an understanding of the discrepancy is still lacking. This leads to two questions:

1a. The authors point out that the difference between the actual and null values of omega_1 changes sign somewhat later than tau_d, and they call this a "regime change." This term is kind of overkill: the sign switch could just as easily be due to the null data decreasing in advance of the peak at tau_m. Moreover, the inset, which is designed to highlight this change, does not add new information, and is in fact misleading: showing a log scale both above and below zero allows one to separate positive and negative values arbitrarily. The authors appear to do just that, making the sign change look like a huge jump. Please remove the inset and tone down the language about the sign change.

We thank the reviewer for raising this fundamental point. These are very much in line with many of the comments made by the other reviewer. As noted above, we agree with the reviewers that the eigenvalue analysis does not clearly pinpoint a pitchfork bifurcation in the data. We have therefore removed the inset and mention of the regime crossover (Results, 4th paragraph of "Covariance analysis pinpoints..", lines 516-518) and more clearly acknowledged the lack of a significant deviation between data and null before T_m (lines 521-523). A more significant deviation is anticipated were the transition a pitchfork style, one-to-many, transition.

Nonetheless, as pointed out by both reviewers, the eigenvector analysis more clearly identifies T_d and T_m as two pseudotime at which something qualitative about the system changes. We have therefore included two new possibilities for the eigenvalue signature at T_d in our manuscript: that it is due to bias in the developmental trajectory toward a neutrophil state, (Results penultimate paragraph of "Covariance analysis pinpoints…", lines 565-570) or that it is a broken pitchfork, as the reviewer suggests in 1b below (lines 548-553 and Supplemental Section S3.3 "Effect of Small Errors", and Fig. S8). Additionally, we have reexamined the eigenvectors at both transition times, to better understand how they differ from each other, by evaluating the fraction of variance for each of their eigenvectors, and their projections onto the developmental tangent vector (Results, third paragraph of "Covariance eigenvectors…", lines 627-642 and Fig. S12) throughout pseudotime as suggested in note 5 above.

1b. It occurred to me that with a pitchfork bifurcation, if the bifurcation trajectory is slightly "off", you do not really get a pitchfork (like an Ising model transitioning through its critical temperature but with a small magnetic field)--you get a "disconnected" pitchfork that is biased to one of the two states. But with a saddle bifurcation, if you are slightly off, you still get a saddle (like an Ising model transitioning through zero magnetic field at two of many subcritical temperatures). This suggests a test in the toy model: if you vary 1/k_D to create the pitchfork, but change one or more the other parameters away from their "ideal" values, does it become harder to distinguish the increase in omega_1 from that in the null model? This would provide mechanistic insight for why a one-to-many transition may be harder to detect via cross-validation than a one-to-one transition.

The Reviewer's comment could not have been more on point. The disconnected, or broken, pitchfork is a very viable candidate for what we might be observing and what might be generic. As the reviewer is no doubt aware, a perfect pitchfork is the consequence of a symmetry (much like the up and down states upon magnetization) in the system. Any variation in parameters that breaks that symmetry breaks the pitchfork. Thus, the broken pitchfork is a far more generic state of affairs than a perfect one. To evaluate the effect of the broken pitchfork on eigenvalue analysis, we have pursued the Reviewer's suggested analyses in our toy model, and incorporated it as a

© 2023. Published by The Company of Biologists under the terms of the Creative Commons Attribution License (https://creativecommons.org/licenses/by/4.0/).

section in the supplement (Supplemental Section "Effect of Small Errors", Figure S3.3). We found that indeed, small errors in other parameters can strongly impact the behavior of the eigenvalue in a one-to-many transition, while playing a much less significant role in the eigenvalue of a one-to-one transition.

This of course raises a fundamental question as to what is the nature of one-to-many transitions we observe in transcriptomic data? While this fascinating question is beyond the scope of our current work, we have incorporated the possibility of an imperfect pitchfork (penultimate paragraph of Results: "Covariance analysis pinpoints..," lines 548-553), as well as the possibility of developmental bias, suggested by the other Reviewer, (lines 565-570) into our interpretation. We believe that the incorporation of these interpretations significantly enhances the biological and theoretical applicability of our analysis and we thank both reviewers for their suggested follow up analyses.

2. The alternative to bifurcation-based state transitions, namely noise-induced state transitions, was not clear to me when it was first introduced (lines 34-38). It only became somewhat clear later when tested with the toy model. I would suggest either adding a cartoon to Fig 1 or adding a sentence or two at those lines to more clearly paint the picture of noise-induced transitions as a plausible mechanism for differentiation.

We agree that as it was stated it left many things unclear. We have included a clarifying statement now in second paragraph of Introduction lines 68-74): "While these studies generally characterize cell fate decisions as bifurcations of an underlying developmental landscape, other studies formulate cell fate transitions as stochastic jumps between coexisting states of a multimodal cell-fate landscape, that can occur even in the absence of bifurcations, to infer lineage relationships and state transition probabilities."

Second decision letter

MS ID#: DEVELOP/2022/201280

MS TITLE: A dynamical systems treatment of transcriptomic trajectories in hematopoiesis

AUTHORS: Simon Freedman, Bingxian Xu, Sidharth Goyal, and Madhav Mani

ARTICLE TYPE: Research Article

I am happy to tell you that your manuscript has been accepted for publication in Development, pending our standard ethics checks. Where referee reports on this version are available, they are appended below.

Reviewer 1

Advance summary and potential significance to field

In this manuscript, the authors seek to identify signatures of cell differentiation dynamics from single-cell RNA sequencing data. Specifically, drawing on synthetic data from a stochastic dynamical model and published experimental data on hematopoietic cell lineages, they show how the local correlation structure of transcriptomic data can reveal bifurcations in the underlying dynamics.

Extracting dynamical models from single-cell data, to move from descriptive analyses of cell trajectories to a more predictive understanding of how they are shaped, and to ask how aptly they are captured by landscape metaphors, is a major challenge. The present study makes a significant step in that direction, and should therefore be received with great interest in the field.

© 2023. Published by The Company of Biologists under the terms of the Creative Commons Attribution License (https://creativecommons.org/licenses/by/4.0/).

Comments for the author

With the additional analyses and changes made to the manuscript, the authors have addressed my concerns, and I can now recommend publication.

Reviewer 2

Advance summary and potential significance to field

I have read through the authors' reply and revised manuscript. All of my comments (and to my judgment, those of the other reviewer) have been addressed sufficiently, and I recommend publication.

Comments for the author

None

© 2023. Published by The Company of Biologists under the terms of the Creative Commons Attribution License (https://creativecommons.org/licenses/by/4.0/).
